# CRISPR/Cas9-assisted gRNA-free one-step genome editing with no sequence limitations and improved targeting efficiency

**DOI:** 10.1038/s41598-017-16998-8

**Published:** 2017-11-30

**Authors:** Dongdong Zhao, Xu Feng, Xinna Zhu, Tao Wu, Xueli Zhang, Changhao Bi

**Affiliations:** 1 Tianjin Institute of Industrial Biotechnology, Chinese Academy of Sciences, Tianjin, 300308 China; 2 Key Laboratory of Systems Microbial Biotechnology, Chinese Academy of Sciences, Tianjin, 300308 China; 3 School of life sciences, China West Normal University, Nanchong, 637002 China; 4 College of Biological Sciences and Technology, Beijing Forestry University, Beijing, 100083 China

## Abstract

The CRISPR/Cas9 system is a powerful, revolutionary tool for genome editing. However, it is not without limitations. There are PAM-free and CRISPR-tolerant regions that cannot be modified by the standard CRISPR/Cas9 system, and off-target activity impedes its broader applications. To avoid these drawbacks, we developed a very simple CRISPR/Cas9-assisted gRNA-free one-step (CAGO) genome editing technique which does not require the construction of a plasmid to express a specific gRNA. Instead, a universal N20 sequence with a very high targeting efficiency is inserted into the *E*. *coli* chromosome by homologous recombination, which in turn undergoes a double-stranded break by CRISPR/Cas9 and induces an intra-chromosomal recombination event to accomplish the editing process. This technique was shown to be able to edit PAM-free and CRISPR-tolerant regions with no off-target effects in *Escherichia coli*. When applied to multi-locus editing, CAGO was able to modify one locus in two days with a near 100% editing efficiency. Furthermore, modified CAGO was used to edit large regions of up to 100 kbp with at least 75% efficiency. Finally, genome editing by CAGO only requires a transformation procedure and the construction of a linear donor DNA cassette, which was further simplified by applying a modular design strategy. Although the technique was established in *E*. *coli*, it should be applicable to other organisms with only minor modifications.

## Introduction

Genome editing technology is used for rationally designed chromosome modification, which is one of the most significant techniques in molecular biology and plays an important role in many biotechnological applications. Current methods include the zinc finger nucleases (ZFNs)^1,2^, the transcription activator-like effector nucleases (TALEN)^3,4^ and the homing meganucleases^5^. However their applications are limited by the need to construct proteins that recognize specific sequences. Recently, an RNA-guided editing system based on CRISPR/Cas9 has provided an alternative genome editing strategy^6^. The CRISPR/Cas9 system is able to recognize and make double-strand breaks (DSBs) at target sequences based purely on nucleotide sequence. Following the DSB event, the native DNA repair process is initiated through non-homologous end joining or homologous recombination. Following modification and simplification, this technique has been harnessed for genome editing in both eukaryotes and prokaryotes, including *E*. *coli* ^7–10^, humans^11,12^, mice^13,14^ and zebrafish^15,16^. Thus, the CRISPR/Cas9 system is already a powerful and revolutionary tool for genome editing.

However, this technology is not without limitations. The binding of Cas9 to DNA is programmed by a single guide RNA (sgRNA)^6^ containing a sequence complementary to a target protospacer, which requires a short neighboring protospacer adjacent motif (PAM) sequence (NGG for Cas9)^6,17,18^. As a result, there are chromosomal regions that cannot be edited by the CRISPR/Cas9 system (PAM-free regions), which are located 20 or more base pairs away from the nearest NGG motif on the chromosome. In addition, single or small-area nucleotide mutations close to the 5′ end of the protospacer greatly reduce CRISPR/Cas9 editing efficiency and accuracy, since they are tolerated by Cas9 to various degrees depending on their positions along the guide RNA - DNA interface^19,20^ (Cas9-tolerant regions). One solution is to evolve the Cas9 protein to surpass the NGG-PAM sequence limitation, and be able to recognize various PAM sequences. This strategy has already had some success, but is not yet a mature solution^21,22^.

The off-target activity of the CRISPR/Cas9 system is probably the most significant problem with this technique, since it can cleave not only the target but also off-target sites that differ by up to several nucleotides^20,23^, causing unwanted mutations and chromosomal rearrangements. One common solution is to thoroughly analyze the entire genome using appropriate software to find N20 sequences with the least possibility to cause off-target effects^24–26^. However, this strategy imposes limitations on available editing loci, and does not guarantee freedom from off-target effects.

To avoid these drawbacks, we designed a very simple CRISPR-based gRNA-free one-step genome editing strategy (CAGO), which requires no gRNA design and construction. Instead, a universal N20 sequence with optimal targeting efficiency is inserted into the chromosome by homologous recombination, which in turn causes a double-strand break via CRISPR/Cas9, and induces an intra-chromosomal recombination event to accomplish the editing process. This technique effectively resolves the sequence limitations of normal CRISPR/Cas9 methods and significantly reduces the off-target frequency.

## Results and Discussion

### Design and development of the CRISPR/Cas9-assisted gRNA-free one-step (CAGO) genome editing technique

**Figure 1:**
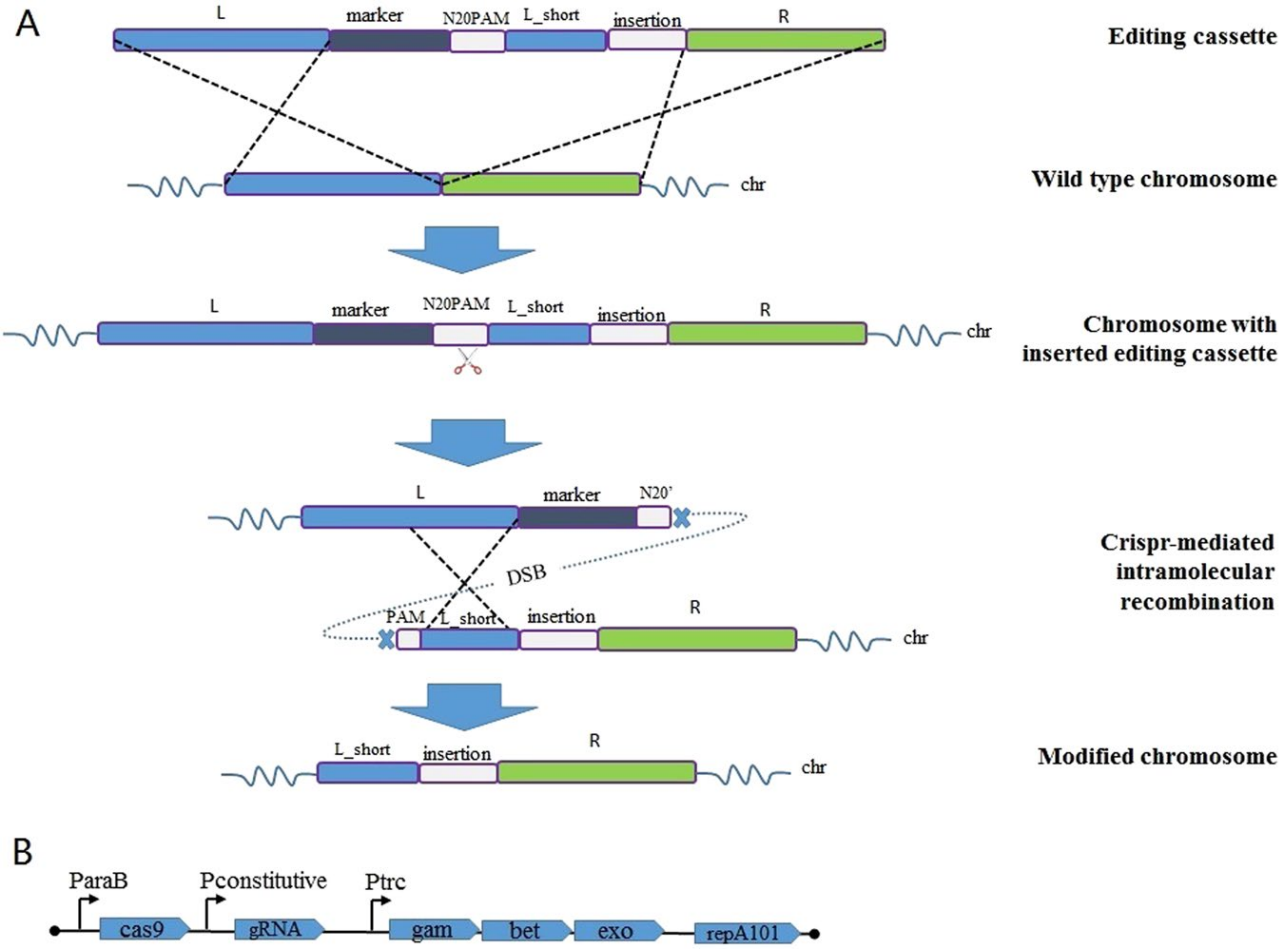
Schematic of the CAGO genome-editing technique and the structure of the pCAGO plasmid. (**A**) The editing cassette is inserted into the target locus by homologous recombination. Subsequently, the CRISPR/Cas9 system is expressed to generate a double-strand break (DSB) at the inserted universal N20PAM sequence, after which a DSB-mediated intra-chromosomal recombination event between the L and L_short homologous arms completes the markerless genome editing. (**B**) Structure of the pCAGO functional region; the λ-Red system is induced using the Ptrc promoter, *cas9* is induced using the pBAD promoter, and gRNA is expressed by a constitutive promoter. The temperature-sensitive repA101ts is used as the plasmid replicon for easy curing afterwards.

A very simple strategy was designed as illustrated in the Fig. 1A. The genome-editing procedure specifically was implemented via a designed DNA-editing cassette, which contained three homology arms of the target locus (L, L_short and R), an insertion fragment if necessary, a selection marker (such as chloramphenicol resistance), and a CRISPR/Cas9 recognition region (N20PAM). This universal N20PAM was designed and selected to have minimal sequence similarity with all N20PAM sequences in the target genome, in order to guarantee the lowest possible off-target frequency. The L_short arm was a shorter 3′-end fragment of the L homologous arm. During the genome editing process, the editing cassette was inserted into a genomic locus via homologous recombination. Subsequently, the CRISPR/Cas9 system was expressed in the cells to generate a DSB at the universal N20PAM site, which induced a DSB-mediated intramolecular recombination event between the two homologous arms - the L_short arm on the editing cassette and L_short on the chromosome, which removed the editing cassette. Thus, a genomic locus was edited with no marker by this simple procedure. This strategy required no gRNA design and construction, since the editing cassette was the only locus-specific item to be constructed.

To facilitate the CAGO technique for genome editing in the model organism *E*. *coli*, the λ-Red system was expressed to increase the efficiency of homologous recombination^27^, which should be controlled according to the expression timing of the CRISPR/Cas9 system. The helper plasmid pCAGO was designed to fulfil this function, in which these two systems were induced separately via the two inducible promoters Ptrc and pBAD (Fig. 1B). The λ-Red system was induced using IPTG both for the insertion of the editing cassette and for intramolecular recombination, while the CRISPR/Cas9 system was induced using L-arabinose in order to generate a DSB at the universal N20PAM to remove the editing cassette. The temperature-sensitive replicon repA101ts was utilized for easy curing of the pCAGO plasmid from the edited cells. A specially designed N20 (5′-TAGTCCATCGAACCGAAGTA-3′) was confirmed to have no complementary sequence in the *E*. *coli* genome using Cas-OFFinder^24^, and was selected as the targeting sequence for inducing a chromosomal DSB with the lowest off-target possibility. There were 9 N20PAM DNA sequences in the genome which were somewhat similar to this selected N20, but each had at least 5 mismatches (Table S3), making them unlikely off-target loci. To experimentally test for any off-target activity of this universal N20PAM sequence, pCAGO was electroporated into *E*. *coli* MG1655 and induced to express its CRISPR/Cas9 system with gRNA targeting the selected N20 sequence. No significant increase of cell death was observed after the induction compared with the control group. For further confirmation, loci containing the most similar sequences were amplified by PCR for sequence analysis. All sequences of the PCR products were proved to be the original *E*. *coli* MG1655 ones, suggesting that no off-target events had happened at any of these loci (Table S3).

### Genome editing of CRISPR-tolerant and PAM-free regions by CAGO with no detected off-target events

**Figure 2:**
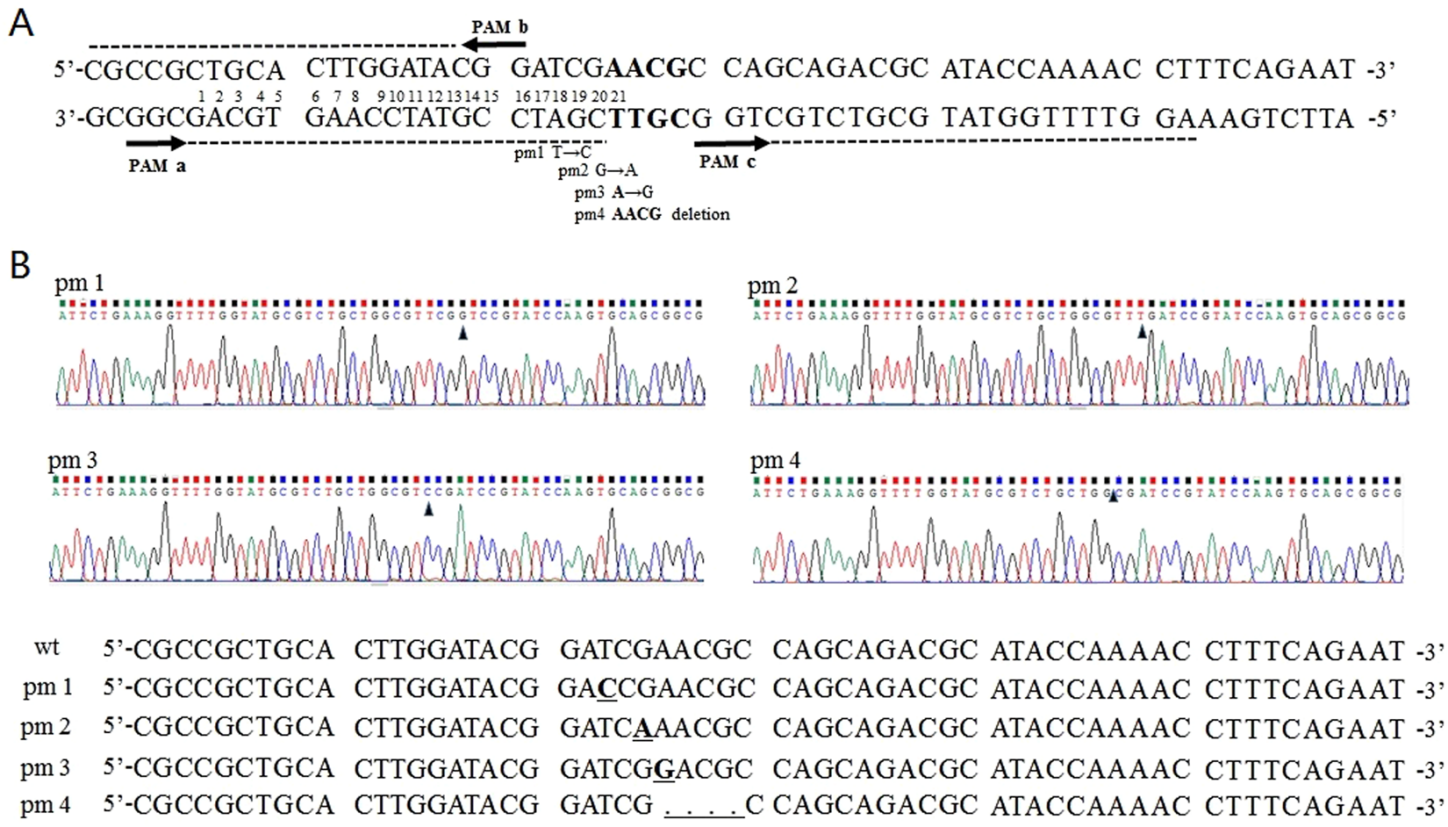
Genome editing events in the CRISPR-tolerant and PAM-free regions within *ldhA*. (**A**) PAM (NGG) sequences are indicated by downward arrows; the dotted lines represent the 20 nt sequences surrounding each PAM; the nucleotides shown in bold that are 20 bp away from any NGG are the PAM-free regions. The nucleotides located from 18 to 20 bp at the 5′ of N20PAM are CRISPR-tolerant regions. Single mismatches in this area may still lead to CRISPR/Cas9-mediated DSBs. ‘pm1’ (T mutated to C, position 18) and ‘pm2’ (G mutated to A, position 20) represent two editing events in the CRISPR-tolerant regions. ‘pm3’ (A mutated to G, position 21) and ‘pm4’ (AACG deletion, bolded area) represent two editing events in the PAM-free regions. (**B**) Original sequencing data of PCR products amplified from the edited area.

As described previously, there are CRISPR-tolerant and PAM-free regions that cannot be edited with current CRISPR-based techniques. To demonstrate the capacity of CAGO to modify such recalcitrant sequences, suitable target loci within the *ldhA* gene were chosen for editing (Fig. 2A). The nucleotides AACG shown in bold located more than 20 bp away from any NGG motif represent a PAM-free region. Within it, an A was mutated to a G at position 21 (pm3) with 90% efficiency, and all four nucleotides were deleted (pm4) with 95% efficiency by the CAGO technique. In the CRISPR-tolerant region of PAM, a T at position 18 was mutated to a C with 95% efficiency (pm1), and a G at position 20 was mutated to an A with 100% efficacy (pm2) (Table 1). In order to research the possibility of intramolecular recombination occurring in a CRISPR/Cas9 independent manner, five transformants after editing cassette transformation were identified by colony PCR and DNA sequencing. As shown in Table 1, there was no obvious intramolecular recombination in the first round of recombination. The efficiency data of the first-round recombination are also shown in Table 1. The sequencing results (Fig. 2B), confirmed the successful editing results. Once again, loci containing sequences most similar to the selected universal N20PAM in the edited strain were amplified by PCR to check for off-target activity. The results indicated no off-target events at these loci (Table S3).

**Table 1:** CAGO editing efficiency in CRISPR-tolerant and PAM-free regions.

Editing event	First-round recombination efficiency (CFU/µg DNA)	Positive colonies/Tested colonies of the first-round recombination	Positive colonies/Tested colonies (%) of the CAGO editing
pm1	244.7 ± 15.5	5/5	38/40 (95%)
pm2	180.3 ± 26.1	5/5	40/40 (100%)
pm3	188.7 ± 34.2	5/5	36/40 (90%)
pm4	225 ± 27.8	5/5	38/40 (95%)

Since there is the possibility of recombination between the L_arm and L_short arm, a special pm1 DNA cassette with no L_short was constructed to compare its recombination efficiency with that of the original pm1 editing cassette. The First-round recombination efficiency of this DNA cassette was determined to be 298.3 ± 27.2 CFU/µg DNA, which was somewhat higher than that of pm1 (244.7 ± 15.5 CFU/µg DNA). This indicated that a small portion of the insertion cassettes may have indeed undergone a recombination between the L-arm and L-short arm, which would have caused a minor reduction of recombination efficiency. Nevertheless, the difference was small enough to be acceptable. In addition, no differences in growth were observed between these two strains.

### Fast and easy editing of multiple loci using CAGO

**Figure 3:**
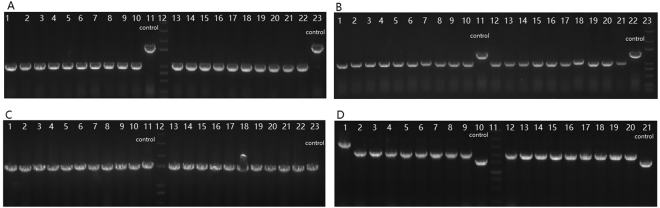
Agarose gel electrophoresis of colony PCR used to confirm editing at multiple loci. (**A**) A *lacZ* gene knockout with a 1018bp deletion: lanes 11 and 23 are control group samples from the original strain, lane 12 is the marker, and the other lanes are experimental group samples from edited strains. Colony PCR products from the edited *lacZ* gene are 982bp, and that of the original is 2000bp long. (**B**) A *poxB* gene knockout with 443bp deletion: lanes 11 and 22 are control group samples, lane 23 is the marker, and the other lanes are experimental group samples. Colony PCR products from edited *poxB* gene are 1083bp, and that of the original is 1520bp long. (**C**) 1018bp of *lac* ***Z*** replaced by a 915bp *kan* fragment: lanes 11 and 23 are control group samples, lane 12 is the marker, and the other lanes are experimental group samples. Colony PCR products of the edited *lacZ* gene are 1897bp, and that of the original is 2000bp long. (**D**) 443bp of ***poxB*** replaced by a 915bp *kan* fragment: lanes 10 and 21 are control group samples, lane 11 is the marker, and the other lanes are experimental group samples. Colony PCR products from the edited *poxB* gene are 1998bp, and that of the original is 1520bp long. Lane 1 had a larger PCR product, indicated one unsuccessful editing event.

*PoxB* and *lacZ* of *E*. *coli* MG1655 were selected as target genes for multi-round genome editing to demonstrate the simplicity of CAGO, since these genes are non-essential and their deletion does not significantly affect cell growth^28,29^. In the deletion experiment, 1018bp was deleted from *lacZ* using the CAGO technique in the first round. Without eliminating the helper plasmid pCAGO, a second round of deletion was performed to delete 443 bp from the *poxB* gene. The editing efficiency of both loci was around 100%. Similarly, two rounds of replacement-editing were performed to insert a 915 bp fragment derived from the *kan* gene into both the *lacZ* and *poxB* loci. A near 100% efficiency was also achieved in both rounds of replacement editing. The efficiencies are shown in Table 2, and gel images of colony PCR are shown in Fig. 3. The transformation with pCAGO took half a day, after which the editing process at one locus took only 1.5 days including the PCR confirmation. The results indicated that CAGO was a fast and easy technique for editing single or multiple loci in *E*. *coli*.

**Table 2:** Efficiency of two rounds of consecutive editing using CAGO.

Editing type	First locus editing			Second locus editing		
	First-round recombination efficiency (CFU/µg DNA)	Positive colonies/Tested colonies of the first-round recombination	Positive colonies/Tested colonies (%) of the CAGO editing	First-round recombination efficiency (CFU/µg DNA)	Positive colonies/Tested colonies of the first-round recombination	Positive colonies/Tested colonies (%) of the CAGO editing
Genomic deletion	198.3 ± 17.6	5/5	Δ*lacZ* (40/40) (100%)	343.7 ± 40.5	5/5	Δ*poxB* (39/40) (95%)
Genomic replacement	251.3 ± 51.2	4/5	*lacZ*::*kan* (39/40) (95%)	382.7 ± 28.3	5/5	*poxB*::*kan* (39/40) (95%)

### Very large genomic deletions were achieved using CAGO

**Figure 4:**
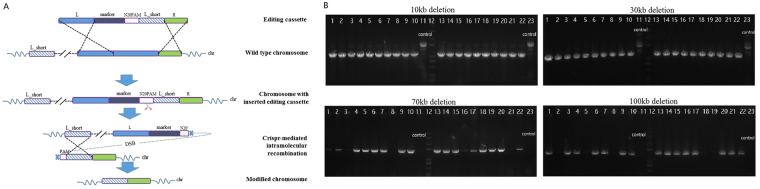
Large genomic deletions using CAGO. (**A**) CAGO technique for large genomic deletions. The L_short arm in the editing cassette was selected to be homologous with a target region upstream of the L homologous arm. When the CRISPR/Cas9 system was expressed to induce the DSB-mediated intramolecular recombination, the L-short region in the editing cassette recombined with its homologous region tens of thousands of bases away, which resulted in a very large chromosomal deletion. (**B**) Agarose gel electrophoresis of colony PCR for the large genomic deletion produced by CAGO. Due to the large size of the PCR-amplified regions, no specific PCR products were seen from the original strains, while specific PCR products were obtained from the successfully edited loci. 10 kb deletion: lanes 11 and 23 are control group samples, lane 12 is the marker, and the other lanes are experimental group samples. Colony PCR products from edited strains are 1139 bp long. 30 kb deletion: lanes 11 and 23 are control group samples, lane 12 is the marker, and the other lanes are experimental group samples. Colony PCR products of the edited type are 1266 bp long. 70 kb deletion: lanes 11 and 23 are control group samples, lane 12 is the marker, and the other lanes are experimental group samples. Colony PCR products of the edited type are 1673 bp long.100 kb deletion: lanes 11 and 23 are control group samples, lane 12 is the marker, and the other lanes are experimental group samples. Colony PCR products of the edited type are 1594 bp long.

To delete or edit very large regions on the chromosome using CAGO, the L_short arm in the editing cassette was designed to be complementary to a target region upstream of the L homologous arm, instead of a shorter 3′-end fragment within the L homologous arm, as illustrated in Fig. 4A. Thus, when the CRISPR/Cas9 system was expressed to induce the DSB-mediated intramolecular recombination, the L-short region in the editing cassette recombined with its complementary region tens of thousands of bases away, which resulted in a very large chromosomal deletion. Genomic segments from 10 kb to 100 kb were chosen for deletion using the CAGO technique, and excellent editing efficiency was achieved as shown in Table 3. A 100% editing efficiency was achieved with deletion of 10 kb and 30 kb fragments, and 75% efficiency was attained for the deletion of regions up to 70 kb and 100 kb. The gel images of corresponding colony PCR products are shown in Fig. 4B.

**Table 3:** Efficiency of large fragment deletion using CAGO.

Targeted genomic region	Deletion size (kb)	First-round recombination efficiency (CFU/µg DNA)	Positive colonies/Tested colonies of the first-round recombination	Positive colonies/Tested colonies (%) of the CAGO editing
b1479-b1489^a^	9.53	319.0 ± 31.2	5/5	40/40 (100%)
b1463-b1489	30.08	279.3 ± 24.0	5/5	40/40 (100%)
b1422-b1489	70.04	399.7 ± 14.5	4/5	32/40 (80%)
b1400-b1489	99.99	446.741.6	5/5	30/40 (75%)

^a^The locus-tags of genes in the genome of *Escherichia coli K-12* (GenBank: U00096.3).

In this research, we developed a very simple technique to edit PAM-free and CRISPR-tolerant genomic regions with low off-target frequency. To the best of our knowledge, this is also the most simple and rapid genome-editing technique in *E*. *coli* thus far. Since the system is gRNA-free, no specific gRNA expression plasmid was necessary in the editing process. Instead, only a linear donor DNA cassette needed to be constructed, which was simplified by using a modular design approach. In the editing process, transformation of pCAGO took half a day, after which the editing process at one locus took only 1.5 days including confirmation by PCR. We have noticed a very recently published article which describes a similar strategy^30^. However, there are some advantages in the CAGO technique compared with previous reports. First, CAGO uses a specifically designed DNA cassette that can induce a DSB-mediated intramolecular recombination event to conduct markerless editing. Thus, only one transformation procedure is needed, which significantly simplifies the editing experiments. Secondly, the well-designed helper plasmid pCAGO provides both the RED recombinase and the CRISPR/Cas9 system controlled by different inducers, which improved the efficiency and simplicity of the editing procedure. Thirdly, the N20 sequence used to eliminate the editing cassette was carefully selected to have the lowest possible probability of inducing off-target effects, thus improving the editing accuracy. Finally, 100 kb or even larger chromosomal fragments could be deleted using the CAGO technique with a very simple procedure, which was not reported before.

Although the CAGO technique was established in the model organism *E*. *coli*, it should be adaptable to other bacteria, simple eukaryotes, and even plant and mammalian cells which have an established expression system and functional homologous recombination efficiency, such as rice^31^ and human cells^32^.

## Methods

### Strains and culture conditions

*E*. *coli* DH5α was used as a cloning host, and the wild-type *E*. *coli* MG1655 was used for the genome editing experiments. MG1655 and DH5α were grown at 30 °C in lysogeny broth (LB; 1% (w/v) tryptone, 0.5% (w/v) yeast extract, 1% (w/v) NaCl). Chloramphenicol (30 mg/L) and ampicillin (100 mg/L; were added to the media where appropriate. 1% (w/v) glucose was added to the cultures to repress the expression of *cas9*, L-arabinose (2g/L final concentration) was used for *cas9* induction, and IPTG (0.1 mM final concentration) was used to induce the λ-Red system.

*E*. *coli* MG1655 cells with or without plasmids were grown in 50-ml of LB medium supplemented with the appropriate antibiotics at 30 °C to an OD_600_ of 0.6, and then made electrocompetent by concentrating 100-fold and washing three times with 10% ice-cold glycerol. 50 ng of plasmid DNA or 800 ng of the editing cassette was used for electroporation. The shocked cells were resuspended in 1 ml of LB, incubated for 2 h at 30 °C, and spread on LB agar plates with the appropriate antibiotics.

### Plasmid construction

The plasmid pCAGO which encodes the inducible expression of λ-Red, Cas9 and gRNA with specifically designed N20 was assembled using the Golden Gate method^33^ in conjunction with DNA oligonucleotides designed using the J5 Device Editor^34^. The λ-Red coding sequence with the Ptrc promoter was amplified by PCR from the plasmids pKD46^35^ and pACYC184-M-crt^36^, while *cas9* and the pBAD promoter were amplified from plasmids pRed_cas9_ΔpoxB300^37^ and plasmid pKD46, respectively. The gRNA was amplified from plasmid pRed_Cas9 with a specifically designed N20 sequences (5′-TAGTCCATCGAACCGAAGTA-3′) embedded within the primers. DNA templates were PCR-amplified using Phusion polymerase (NEB), the PCR products were purified from a 1% agarose gel after electrophoresis, and digested using *Dpn*I (NEB) for Golden Gate assembly. All primers used for plasmid construction are listed in Table S1.

### Construction of the editing cassette

**Figure 5:**
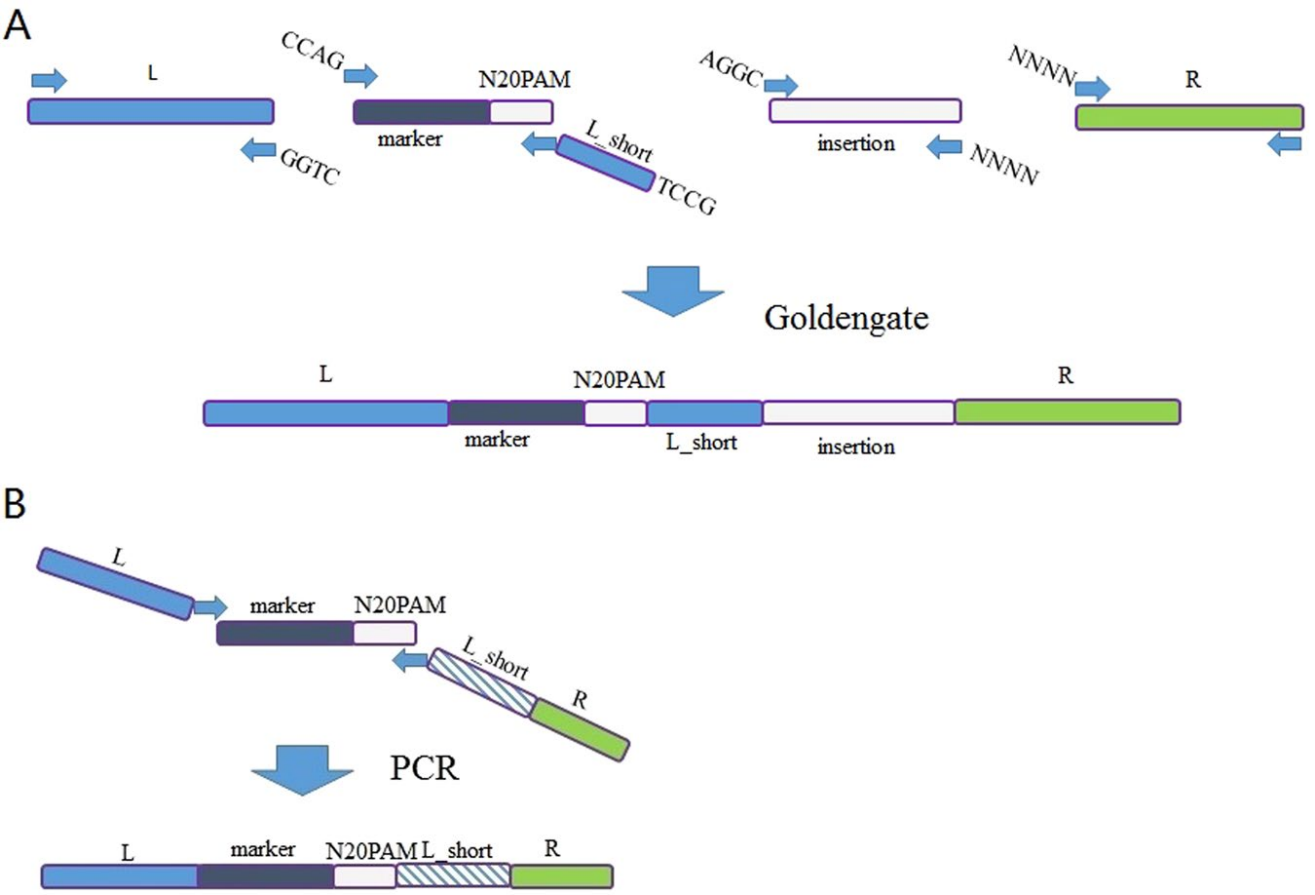
Modularized construction of the editing cassette. (**A**) Four modular parts were prepared with optimized 4-nt linkers that can be processed using type IIS restriction enzymes for assembly, including a left homologous arm (L), a selection marker - N20PAM, an L_short arm, an insertion fragment and a right homologous arm (R). A Golden Gate reaction was preformed to assemble these parts into the editing cassette. (**B**) For genomic deletions, the construction of the editing cassette was simplified to one PCR reaction. The left homologous arm (L) of 65bp was embedded within the forward primer, while the right homologous arm (R, 40bp) and L_short (40 bp) arm were embedded within the reverse primer. The L_short sequence was taken from the region upstream of the left homologous arm.

Four modularized parts were prepared with optimized 4-nt linkers that can be processed using type IIS restriction enzymes for assembly (Fig. 5A), which included a left homologous arm (L), a selection marker (Chloramphenicol resistance gene, Cm) with a CRISPR/Cas9 recognition region (N20PAM), an L_short arm, an insertion fragment, and a right homologous arm (R). The L_short arm was a key element designed to be 40bp long. With this length, it was short enough to be embedded within a PCR primer for easy construction, while it was still long enough to confer good editing efficiency. In the deletion experiments, three parts were necessary, whereby L_short was embedded in the forward primer of the right homology arm (R) or the reverse primer of the selection part. A Golden Gate reaction was preformed to assemble these parts into the editing cassette. The optimized 4bp type IIS restriction linkers were tested for high assembly efficiency, and the linker between the insertion fragment and the right homology arm was variable. The L and R homologous arms (about 500bp each) were amplified from the genomic DNA of *E*. *coli* MG1655. The selection marker with the CRISPR/Cas9 recognition region (N20PAM) was PCR-amplified from plasmid pACYC184-M-crt with the N20PAM sequence embedded in the reverse primer, and was used as template for PCR amplification to add type IIS linkers.

For genomic deletions, the editing cassette was constructed by PCR amplification without Golden Gate assembly. In this case, the left homologous arm (L) with a length of 65bp was embedded within the forward primer, and the right homologous (R) and L_short arms with a length of 40bp were embedded within the reverse primer. The L_short sequence was obtained from the region upstream of the left homologous arm (Fig. 5B). All primers used for the construction of editing cassettes are listed in Table S4.

### Genome editing procedure

**Figure 6:**
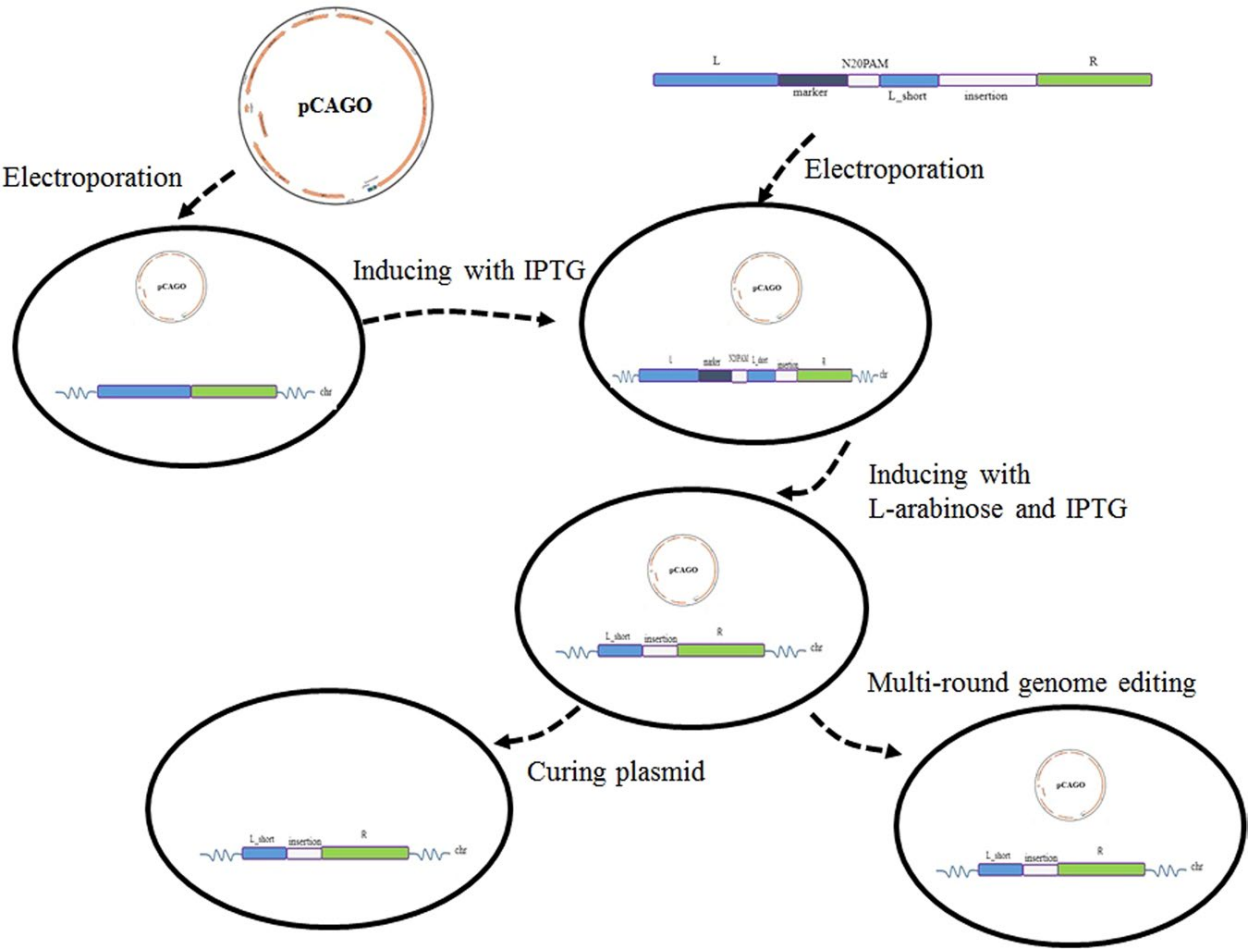
The procedure for genome editing with CAGO. *E*. *coli* cells transformed with pCAGO were induced with IPTG to express the λ-Red system, and electroporated with the editing cassette. After the editing cassette was inserted at the target locus, both IPTG and L-arabinose were added to the cultures to induce the expression of λ-Red and CRISPR/Cas9, which facilitated the removal of the editing cassette and yielded the final edited locus. The helper plasmid pCAGO can be cured by culturing overnight at 42 °C or left in the cells for the next round of editing.

The genome editing process is illustrated in Fig. 6. *E*. *coli* MG1655 competent cells harboring pCAGO were prepared with λ-RED proteins expressed by IPTG induction. An aliquot comprising 50 μL of the competent cells was mixed with 800 ng of editing-cassette DNA in a 2-mm Gene Pulser cuvette (Bio-Rad). After electroporation at 2.5 kV and immediate resuspension in 1 ml of ice-cold LB medium, the cells were incubated for 2 hours at 30 °C, and then spread on LB agar plates with ampicillin, chloramphenicol and 1% glucose. For each editing experiment, five transformants were identified by colony PCR with a forward primer complementary to the selection marker and a reverse primer downstream of the right homology arm, and the expected PCR products were subjected to DNA sequencing for further confirmation. A correct clone was transferred into LB media with ampicillin, IPTG, and L-arabinose to induce the expression of the CRISPR/Cas9 system and λ-RED protein. After cultivation at 30 °C for more than 6 hours, the cells were spread on LB agar plates with ampicillin. To identity correctly edited clones, 40 colonies were analyzed by colony PCR with a forward primer that binds upstream of the left homology arm and a reverse primer that binds downstream of the right homology arm. Ten colonies with the expected PCR products were subjected to DNA sequencing for further confirmation. Since all the sequenced PCR products turned out to have the expected edited genotype, other colonies with such PCR products were considered to also be successfully edited clones. The editing efficiency was calculated as the number of colonies with correct PCR product divided by the number of tested colonies. For continuous editing of additional genomic loci, the correct clones were used as parent strains for transformation of new editing DNA cassettes. After obtaining all genome modifications, the editing plasmid was cured by growing overnight at 42 °C. A detailed genome editing protocol is provided in the supplemental file.

## Electronic supplementary material

Supplementary Information

## References

[1] Bibikova M, Golic M, Golic KG, Carroll D (2002). Targeted chromosomal cleavage and mutagenesis in Drosophila using zinc-finger nucleases. Genetics.

[2] Urnov FD, Rebar EJ, Holmes MC, Zhang HS, Gregory PD (2010). Genome editing with engineered zinc finger nucleases. Nature Reviews Genetics.

[3] Miller JC (2011). A TALE nuclease architecture for efficient genome editing. Nature biotechnology.

[4] Mahfouz MM (2011). De novo-engineered transcription activator-like effector (TALE) hybrid nuclease with novel DNA binding specificity creates double-strand breaks. Proceedings of the National Academy of Sciences.

[5] Stoddard BL (2005). Homing endonuclease structure and function. Quarterly Reviews of Biophysics.

[6] Jinek M (2012). A Programmable Dual-RNA-Guided DNA Endonuclease in Adaptive Bacterial Immunity. Science.

[7] Jiang W, Bikard D, Cox D, Zhang F, Marraffini LA (2013). RNA-guided editing of bacterial genomes using CRISPR-Cas systems. Nature biotechnology.

[8] Jiang Y (2015). Multigene Editing in the Escherichia coli Genome via the CRISPR-Cas9 System. Applied and environmental microbiology.

[9] Li Y (2015). Metabolic engineering of Escherichia coli using CRISPR–Cas9 meditated genome editing. Metabolic engineering.

[10] Chung, M. E. *et al*. Enhanced integration of large DNA into E. coli chromosome by CRISPR/Cas9. *Biotechnology & Bioengineering* (2016). 10.1002/bit.26056 27454445

[11] Cho SW, Kim S, Kim JM, Kim JS (2013). Targeted genome engineering in human cells with the Cas9 RNA-guided endonuclease. Nature Biotechnology.

[12] Shalem O (2014). Genome-scale CRISPR-Cas9 knockout screening in human cells. Science.

[13] Wang H (2013). One-Step Generation of Mice Carrying Mutations in Multiple Genes by CRISPR/Cas-Mediated Genome Engineering. Cell.

[14] Fujii W, Kawasaki K, Sugiura K, Naito K (2013). Efficient generation of large-scale genome-modified mice using gRNA and CAS9 endonuclease. Nucleic acids research.

[15] Chang N (2013). Genome editing with RNA-guided Cas9 nuclease in zebrafish embryos. Cell research.

[16] Hwang WY (2013). Efficient genome editing in zebrafish using a CRISPR-Cas system. Nature Biotechnology.

[17] Mojica FJM, Díezvillaseñor C, Garcíamartínez J, Almendros C (2009). Short motif sequences determine the targets of the prokaryotic CRISPR defence system. Microbiology.

[18] Shah SA, Erdmann S, Mojica FJ, Garrett RA (2013). Protospacer recognition motifs: mixed identities and functional diversity. Rna Biology.

[19] Anderson EM (2015). Systematic analysis of CRISPR–Cas9 mismatch tolerance reveals low levels of off-target activity. Journal of Biotechnology.

[20] Fu Y (2013). High-frequency off-target mutagenesis induced by CRISPR-Cas nucleases in human cells. Nature biotechnology.

[21] Kleinstiver, B. P. *et al*. Broadening the targeting range of Staphylococcus aureus CRISPR-Cas9 by modifying PAM recognition. *Nature Biotechnology* **33** (2015). 10.1038/nbt.3404 PMC4689141 26524662

[22] Kleinstiver BP (2015). Engineered CRISPR-Cas9 nucleases with altered PAM specificities. Nature.

[23] Cho SW (2014). Analysis of off-target effects of CRISPR/Cas-derived RNA-guided endonucleases and nickases. Genome research.

[24] Bae S, Park J, Kim JS (2014). Cas-OFFinder: a fast and versatile algorithm that searches for potential off-target sites of Cas9 RNA-guided endonucleases. Bioinformatics.

[25] Heigwer F, Kerr G, Boutros M (2014). E-CRISP: fast CRISPR target site identification. Nature Methods.

[26] Naito Y, Hino K, Bono H, Ui-Tei K (2015). CRISPRdirect: software for designing CRISPR/Cas guide RNA with reduced off-target sites. Bioinformatics.

[27] Yu D, Ellis HM, Lee E-C, Jenkins NA, Copeland NG (2000). An efficient recombination system for chromosome engineering in Escherichia coli. Proceedings of the National Academy of Sciences.

[28] De Mey M, De Maeseneire S, Soetaert W, Vandamme E (2007). Minimizing acetate formation in E. coli fermentations. Journal of industrial microbiology & biotechnology.

[29] Wang HH (2009). Programming cells by multiplex genome engineering and accelerated evolution. Nature.

[30] Zhang, H., Cheng, Q. X., Liu, A. M., Zhao, G. P. & Wang, J. A Novel and Efficient Method for Bacteria Genome Editing Employing both CRISPR/Cas9 and an Antibiotic Resistance Cassette. *Frontiers in Microbiology* **8** (2017). 10.3389/fmicb.2017.00812 PMC5418352 28529507

[31] Terada R, Urawa H, Inagaki Y, Tsugane K, Iida S (2002). Efficient gene targeting by homologous recombination in rice. Nature Biotechnology.

[32] Zwaka TP, Thomson JA (2003). Homologous recombination in human embryonic stem cells. Nature Biotechnology.

[33] Engler C, Kandzia R, Marillonnet S (2008). A one pot, one step, precision cloning method with high throughput capability. PloS one.

[34] Hillson NJ, Rosengarten RD, Keasling JD (2011). j5 DNA assembly design automation software. ACS Synthetic Biology.

[35] Datsenko KA, Wanner BL (2000). One-step inactivation of chromosomal genes in Escherichia coli K-12 using PCR products. Proceedings of the National Academy of Sciences.

[36] Zhao J (2013). Engineering central metabolic modules of Escherichia coli for improving β-carotene production. Metabolic engineering.

[37] Zhao, D. *et al*. Development of a fast and easy method for Escherichia coli genome editing with CRISPR/Cas9. *Microbial Cell Factories* **15** (2016). 10.1186/s12934-016-0605-5 PMC5134288 27908280

