# Supplementary material for: CRISPR/Cas9-assisted gRNA-free one-step genome editing with no sequence limitations and improved targeting efficiency
Source: Sci Rep. 2017 Nov 30;7:16624. doi: 10.1038/s41598-017-16998-8 (PMC5709385; doi:10.1038/s41598-017-16998-8)
Supplement: Supplementary file 1 — Supplementary Information [file 41598_2017_16998_MOESM1_ESM.pdf]

## Supplementary Information

**CRISPR assisted gRNA free one-step genome editing technique with no sequence limitations and improved targeting efficiency**

Dongdong Zhao, Xu Feng, Xinna Zhu, Tao Wu, Xueli Zhang & Changhao Bi

# Figure

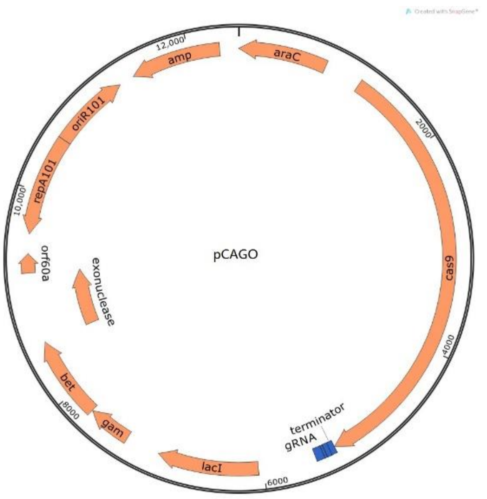


Figure S1. pCAGO map

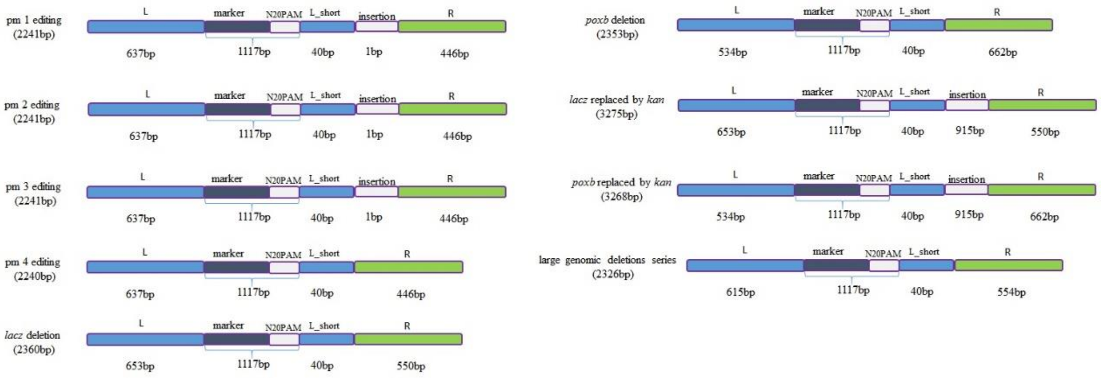


Figure S2. Editing cassettes with labeled by size

# Table

Table S1. Primers for construction of pCAGO

| Name | Sequence |
|---|---|
| (pkd46)_forward | CCAGGTCTCACCATGGATATTAATACTGAAACTGAGATCAAGC |
| (pkd46)_reverse | CCAGGTCTCAATTTTTTATAACCTCCTTAGAGCTCGAATTCCC |
| (cas9)_forward | CCAGGTCTCAAAATGGATAAGAAATACTCAATAGGCTTAGATATCGGC |
| (cas9)_(N20A)_reverse | CCAGGTCTCATTCGGTTCGATGGACTAGCTAAGATCTGACTCCATAACAGAGTACTCGCC |
| (N20B)_(gRNA)_forward | CCAGGTCTCACGAAGTAGTTTTAGAGCTAGAAATAGCAAGTTAAAATAAGGC |
| (gRNA)_reverse | CCAGGTCTCAGGAGAGAACATACTGGCTAAATACGGAAGG |
| (ptrc)_forward | CCAGGTCTCACTCCATACGATATAAGTTGTAATTCTCATGTTTG |
| (ptrc)_reverse | CCAGGTCTCAATGGTATATCTCCTTGAATTCCATGGTC |

Table S2. Primers used for analysis of chromosomal areas most similar to the slected N20PAM [a]

| Primer Name | Sequence |
|---|---|
| N1G_F | GATTTGCCGCTGTCAAAC |
| N1G_R | ATCACCGCGAAAGTGAAG |
| N2G_F | GGCGGCATTCGTATCGAAGAC |
| N2G_R | TGGCCGATGGCATCAAAGG |
| N3G_F | CACCAGCATTGTCGGTTTAC |
| N3G_R | AAGCCGTGAAGGAGTGAAAG |
| N4G_F | CGCGTTAATCACGTCTCTATCG |
| N4G_R | TTTGATCTGGAGGCGCTAAAGG |
| N5G_F | TTCACGGAATGCGGCAACC |
| N5G_R | AGCGAACGAAGCGGAACTG |
| N6G_F | GGAACACCAGGCGATGATGTC |
| N6G_R | GCACCACCTGCTGTATGATCC |
| N7G_F | ATGGCAGGCCAGAATGAC |
| N7G_R | CTGCTGCTGCAACTTCAC |
| N8G_F | AAACCCGTCTGGTTCATACCC |
| N8G_R | GCCCTTCACCCAGAATATAAGC |
| N9G_F | GGATGATGTTGGCTTCAG |
| N9G_R | TACGGCTTCGATATCACC |

[a] : Choromosomal areas most similar to selected N20PAM were predicted by Cas-OFFinder

Table S3. N20PAM similar areas [a] seqeunces and analysis result after Cas9/gRNA expression

| number | similar DNA sequences | Positions[b] | Mismatches | Positive colonies[c]/Tested | |
|---|---|---|---|---|---|
| | | | | pCAGO exp[d] | Pm4 exp[e] |
| 1 | cAGaCCAcCaAACCcAAGTgCGG | 29456 | 6 | 10/10 | 10/10 |
| 2 | TAGTtCtgCaAAaCGAAGTgGGG | 608522 | 6 | 10/10 | 10/10 |
| 3 | TgGcCaATCaAACaGAAaTATGG | 507357 | 6 | 10/10 | 10/10 |

| | | | | | |
|---|---|---|---|---|---|
| 4 | TAaaCCATCtAcCgGAAtTACGG | 1067543 | 6 | 10/10 | 10/10 |
| 5 | TtcgCCATtGAACtGAAcTAAGG | 1398303 | 6 | 10/10 | 10/10 |
| 6 | TgGTtCATCaAACCGcgGaAGGG | 1580707 | 6 | 10/10 | 10/10 |
| 7 | TAtTaCAcCGAtCCGAAGaATGG | 2247074 | 5 | 10/10 | 10/10 |
| 8 | gAGTCtATCtAtCtGAAcTATGG | 3700191 | 6 | 10/10 | 10/10 |
| 9 | TAtgtaATCaAACCGAAaTACGG | 4619275 | 6 | 10/10 | 10/10 |

[a] : Choromosomal areas most similar to selected N20PAM were predicted by Cas-OFFinder

[b] : Genome positions of *Escherichia coli* (*K-12, MG1655*) predicted by Cas-OFFinder

[c] : Strains with wild type *E. coli* MG1655 seqeunce identified with PCR amplified and DNA sequencing

[d] : N20PAM Similar areas were PCR amplified and sequenced to check off-target possibility after expression pCAGO

[e] : N20PAM Similar areas were PCR amplified and sequenced to check off-target possibility after editing editing experiment pm4

Table S4 Primers for construction of editing cassettes and colony PCR identification

| Primers | Sequence | Characteristics |
|---|---|---|
| N20PAM_Cm_F | TTCATGTGCAGCTCCATCACTGGAGCACCTCAAAAACACCA | Selection marker - N20PAM part amplification |
| N20PAM_Cm_R | CAACGTCATCTCGTTCTCCGCTTATTTGTTAACTGTTAATTGTCCTTACTTCGGTTCGATGGACTATTACGCCCCGCCCTGCCA | |
| Large_L500F | CCACGGCTGTGATTAGAAC | |
| Large_ ReN20PAM2_L500R | CCAGGTCTC**ctgg**TACCCGCCGAAGAAATTCC | |
| ReN20PAM2BsaI_F | CCAGGTCTCA**ccag**TTCATGTGCAGCTCCATC | |
| ReN20PAM2_large_BsaI_R | CCAGGTCTCA**aggc**CAACGTCATCTCGTTCTC | |
| large_L10K _ BSAI_R500F | CCAGGTCTCA**gcct** acacgccgcgtgaacggcggtagatctcagaaaaacgctcGCGATTCGTTGAGTACAG | |
| large_L30K _ BSAI_R500F | CCAGGTCTCA**gcct** tatatgctgtgatgcaagaacagtttggtctgggcgtggaGCGATTCGTTGAGTACAG | |
| large_L70K _ BSAI_R500F | CCAGGTCTCA**gcct** ttacgatgaagcgaccgtccggcagtgttgatggcgtcaaGCGATTCGTTGAGTACAG | |
| large_L100K _ BSAI_R500F | CCAGGTCTCA**gcct** ccaaagcagaaatgccagctaattacctgattgtcggcagGCGATTCGTTGAGTACAG | |
| large_R500R | TGCGCTATCGAAAGTGAG | Large genome deletion |
| Large_genome_R | TAGATGCGCCAGGATGCAG | |
| Large_genome_10k_F | ACCACCGGAAGGGTATACG | |
| Large_genome_30k_F | CGCGCCGATTCGTTTAGTTTC | |
| Large_genome_70kA_F | GAAGAGAACGTCGCATCTAC | |
| Large_genome_70kA_R | CGAAGATATTGCCCTCTCAC | |
| Large_genome_100kA_F | GCGCGTCAGCTGTGTATCAAC | |
| Large_genome_100kA_R | TGCCCTCTCACTTTCCATCGG | |
| Poxb_L500F | CCCTCCGTCAGATGAACTAAAC | |
| Poxb_L500BSAI_R | CCAGGTCTCA**CTGG**TAACACGACAACCGAAAC | |
| ReN20PAM2BSAI_F | CCAGGTCTCA**CCAG**TTCATGTGCAGCTCCATC | |
| ReN20PAM2_poxb_BSAI_R | CCAGGTCTCA**gcCT**GACAACCGAAACGCCACGGTTAAGCACCGCTTTGCGCATGCAACGTCATCTCGTTCTC | |
| Poxb_R500BSAI_F | CCAGGTCTCA**aGGC**ACTGGTCGGCGATATCAAG | *Poxb* editing |
| Poxb_R500R | CGACCACCACATCCACCAACAC | |
| kan_re_poxbfullF | CCAGGTCTCA**aggc**CGTCGGAATTGCCAGCTGGG | |
| kan_re_fullR | CCAGGTCTCA**agtc**TCAGAAGAACTCGTCAAGAAGGC | |
| poxb_kan_R500BSAI_F | CCAGGTCTCA**gact**ACTGGTCGGCGATATCAAG | |

| | | |
|---|---|---|
| poxb_genome _F | CGCCTTATGCCCGATGATATTC | |
| poxb_genome _R | CCAGCACGCTGTTGTTAAAGAC | |
| lacz_L500F | CTTCCGGCTCGTATGTTG | |
| lacz_L500BSAI_R | CCAGGTCTCA**CTGG**GATAACTGCCGTCACTCC | |
| ReN20PAM2BSAI_F | CCAGGTCTCA**CCAG**TTCATGTGCAGCTCCATC | |
| ReN20PAM2_lacz_BSAI_R | CCAGGTCTCA**GCCT**CCGGCGCGTAAAAATGCGCTCAGGTCAAATTCAGACGGCACAACGTCATCTCGTTCTC | |
| lacz_R500BSAI_F | CCAGGTCTCA**AGGC**TGGCTTTCGCTACCTGGAGAG | |
| lacz_R500R | TCGCGTTCGGTTGCACTAC | *Lacz* editing |
| kan_re_laczfullF | CCAGGTCTCA**cgctC**GTCGGAATTGCCAGCTGGG | |
| kan_re_fullR | CCAGGTCTCA**agtc**TCAGAAGAACTCGTCAAGAAGGC | |
| lacz_kan_R500BSAI_F | CCAGGTCTCA**gact**GGCTTTCGCTACCTGGAGAG | |
| lacZ_genome_F | AAAACCCTGGCGTTACCCA | |
| lacZ_genome_R | CAGGCAGTTCAATCAACTGTTTACC | |
| ldhApm_L500F | TACTTACACATCCCGCCATCAGCAGG | |
| ldhApm_L500BSAI_R | CCAGGTCTCA**CTGG**TCCGTATCCAAGTGCAGCGGCG | |
| ReN20PAM2BSAI_F | CCAGGTCTCA**CCAG**TTCATGTGCAGCTCCATC | |
| ReN20PAM2_ldhA_BSAI_R | CCAGGTCTCA**TCCG**TATCCAAGTGCAGCGGCGCTGGAACTCGGTGTGGAGCAACGTCATCTCGTTCTC | |
| ReN20PAM2_ldhAS_BSAI_R | CCAGGTCTCATCCGCAACGTCATCTCGTTCTC | |
| ldhApm1_R500BSAI_F | CCAGGTCTCA**CGGA**Ccg aacg CCAGCAGACGCATACCAAAACC | CRISPR-tolerant and PAM-free |
| ldhApm2_R500BSAI_F | CCAGGTCTCA**CGGA**tcA aacg CCAGCAGACGCATACCAAAACC | regions editing |
| ldhApm3_R500BSAI_F | CCAGGTCTCA**CGGA**tcg Gacg CCAGCAGACGCATACCAAAACC | |
| ldhApm4_R500BSAI_F | CCAGGTCTCA**CGGA**tcgCCAGCAGACGCATACCAAAACC | |
| ldhApm_R500R | CAACAGGTGAACGAGTCCTTTGGC | |
| ldhA_genome_F | TTAGCGCACATCATACGGGTC | |
| ldhA_genome_R | GCGCCTACACTAAGCATAGTTG | |
| Poxb_L65F | ccgcgaatgtagtcactattgcgagctggtttccagcccggagcagatcccacaagtactggcgaTTCATGTGCAGCTCCATC | *Poxb* deletion using simple |
| Poxb_R40L40QR | CTTTTTCTTCCACCAATGGAAGCAATGCACGCAGAGTCGA**AATAGCTCTTGTGGGTGGGTTTCCTGGAAATAGCCGCTGC**CAACGTCATCTCGTTCTC | editing cassette construction strage |

## CAGO technique protocol

## Step1: Editing cassette construction

(1) Amplification of modularized parts

The selection marker part with CRISPR/Cas9 recognition region (N20PAM) are PCR-amplified from plasmid pACYC184-M-crt with N20PAM sequence embedded in reverse primer, which is used as template for PCR amplification to add type IIS linkers. Primer pairs are designed as: 5'..TTCATGTGCAGCTCCATCACTGGAGCACCTCAAAAACACCA..3' and 5'..CAACGTCATCTCGTTCTCCGCTTATTTGTTAACTGTTAATTGTCCTTACTTCGGTTCGATGGACTATTACGCCCCGCCCTGCCA..3'.

For genome deletion, three modularized parts are used for editing cassette construction. The primers used for amplification of each part are in the following table.

Table S5. Primers designed for modularized parts amplification in genome deletion experiment

| modularized parts | Primers design |
|---|---|
| left homology arm (L) | 5'.. 18~22nt forward primer..3' |
| | 5'..CCAGGTCTCACTGG-18~22nt reverse primer..3' |
| marker with N20PAM | 5'..CCAGGTCTCACCAGTTCATGTGCAGCTCCATC..3' |
| | 5'..CCAGGTCTCANNNN-40nt L_short-CAACGTCATCTCGTTCTC..3'[a] |
| right homology arm (R) | 5'..CCAGGTCTCANNNN-18~22nt reverse primer..3' |
| | 5'.. 18~22nt reverse primer ..3' |

[a] :The 40nt L_short could be any DNA fragment upstream of L homologous arm as necessary.

[b] : “NNNN” was the 4 nt linkers between L_short and R, was alterable as needed.

For genome insertion and replacement, four modularized parts are used for editing cassette construction, the primers used for amplification of each part are in the following table.

Table S6. Primers designed for modularized parts amplification in genome insertion and replacement experiment

| modularized parts | Primers design |
|---|---|
| left homology arm (L) | 5'.. 18~22nt forward primer..3' |
| | 5'..CCAGGTCTCACTGG-18~22nt reverse primer..3' |
| marker with N20PAM | 5'..CCAGGTCTCACCAGTTCATGTGCAGCTCCATC..3' |
| | 5'..CCAGGTCTCAGCCT-40nt L_short-CAACGTCATCTCGTTCTC..3' [a] |
| insertion | 5'..CCAGGTCTCAAGGC-18~22nt forward primer..3' |
| | 5'..CCAGGTCTCANNNN-18~22nt reverse primer..3' [b] |
| right homology arm (R) | 5'..CCAGGTCTCANNNN-18~22nt reverse primer..3' |
| | 5'.. 18~22nt reverse primer ..3' |

[a] :The 40nt L_short could be any DNA frsgmrnt upstream of L homologous arm as necessary.

[b] : “NNNN” was the 4 nt linkers between insertion part and R, was alterable as needed.

(2) Modular parts assembly

Add around 100 ng of marker N20PAM part and equal molar amounts of the other assembly parts into a 15 μl total volume assembly reaction mixture:

marker N20PAM part (100 ng)

+ each additional assembly parts (to equal molar with marker with N20PAM part)

+ 1.5 µl 10X NEB T4 Buffer

+ 0.15 µl 100X BSA

+ 1 µl BsaI

+ 1 µl NEB T4 Ligase, 2 million cohesive end units / mL

+ dH20 to 15 µl

Perform the assembly reaction in a thermocycler with following condition:

3 min @ 37 C }

4 min @ 16 C } 25 cycles

5 min @ 50 C }

5 min @ 80 C } 1 cycle

Forward primer of L and reverse primer of R are used for the amplification from the assembly reaction to get large number of editing cassette.

For genome deletion, the editing cassette could also be constructed by PCR amplification without assembly. The left homology arm (L) is embedded in the forward primer with a length of about 65bp, the right homology arm (R) and L_short are embedded in the reverse primer with the length of 40bp for each. Two primers are designed as followed:

5'..65nt left homology arm-TTCATGTGCAGCTCCATC..3' and

5'..40nt reverse right homology arm-40nt reverse L_short-CAACGTCATCTCGTTCTC..3'. The PCR product is the editing cassette for genome editing.

**Step2: Procedure for genome editing**

MG1655 competent cells harboring pCAGO are prepared with IPTG induced λ-RED proteins. 50 µL competent cells are mixed with 400 ng editing cassette in a 2-mm Gene Pulser cuvette (Bio-Rad). After electroporation at 2.5 kV and suspended immediately in 1 ml of ice cold medium, cells are incubated for 2 hours at 30°C, and then plated on solid LB medium with ampicillin, chloramphenicol and 1% glucose.

For each editing experiment, five transformants are analyzed by colony PCR. A correct clone with inserted editing cassette is inoculated into LB media with ampicillin, IPTG, and L-arabinose for CRISPR/Cas9 system and λ-RED protein expression. After culturing for more than 6 hours, cells are plated on LB agar plates with ampicillin. Colonies are identified by colony PCR and the correct colonies are used for further verification by DNA sequencing.

**Step3: Plasmid curing and multiple rounds of genome editing**

In edited strain, pCAGO is cured by growing overnight at 42°C. For consecutive editing, edited clone carrying pCAGO is used as parent strain for transformation of new editing DNA cassette.

## Sequencing data

Deletion of 9.53kb:

TGCCGGTTCTGGTAAGAGATAAACACGCCGCGTGAACGGCGGTAGATCTCAGAAAAACGCTCGCGA
TTCGTTGAGTACAGATTCGATGTTACGACAAATGATTTCCCCCATTTCATGAAATGGC

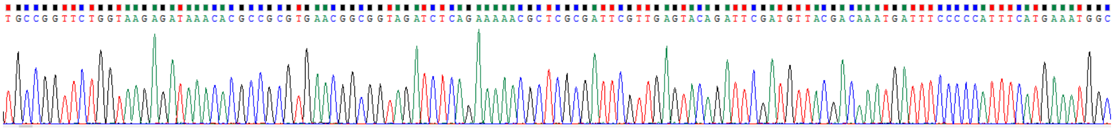

Deletion of 30.08kb:

GTGGAGCAGCGAAATCTACCGGATGTGGCGTCATTATATGCTGTGATGCAAGAACAGTTTGGTCTG
GGCGTGGAGCGATTCGTTGAGTACAGATTCGATGTTACGACAAATGATTTCCCCCATTTCATGAAAT
GGCGGGCTGCTGCACATGGC

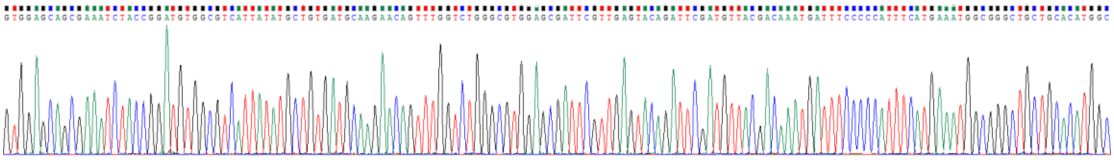

Deletion of 70.04kb:

GCACCAACCCTGACGACATCATTATTAAGACCTTCTTTACGATGAAGCGACCGTCCGGCAGTGTTGAT
GGCGTCAAGCGATTCGTTGAGTACAGATTCGATGTTACGACAAATGATTTCCCCCATTTCATGAAATG
GCGGGCTGCT

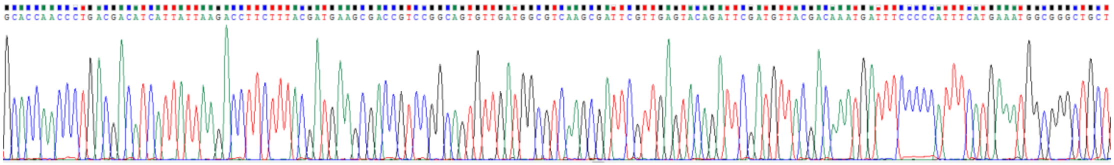

Deletion of 99.99kb:

CGAGAACAGCATTGTTGGTGCATCCGCATTTGTGAAAGCCAAAGCAGAAATGCCAGCTAATTACCTG
ATTGTCGGCAGGCGATTCGTTGAGTACAGATTCGATGTTACGACAAATGATTTCCCCCATTTCATGAA

ATG

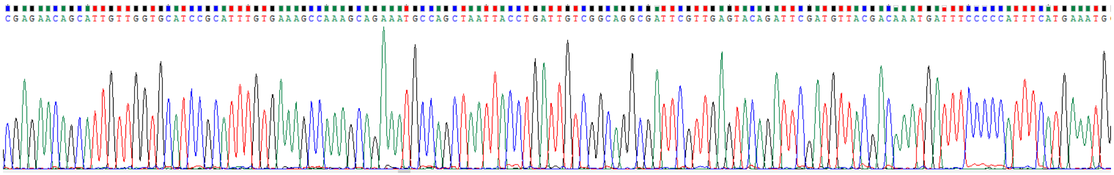

**_Lacz_ deletion:** 1018bp was deleted from the *lacz* gene

CATCGCGTGGGCGTATTCGCAAAGGATCAGCGGGCGCGTCTCTCCAGGTAGCGAAAGCCAGCGGTT
TTCTCCGGCGCGTAAAAATGCGCTCAGGTCAAATTCAGACGGCAAACGACTGTCCTGGCCGTAACCG

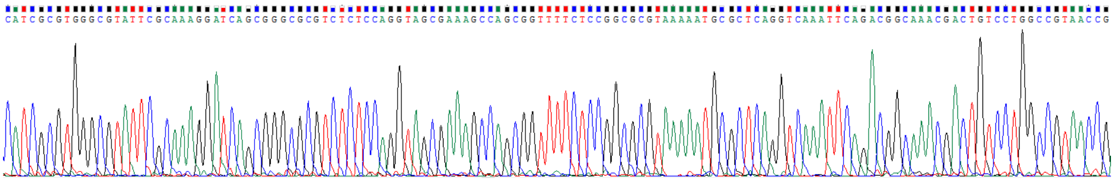

**_Poxb_ deletion:** 443 bp was deleted from the *poxb* gene

CGCTTTATCCAGAAACTTGCGATCGGCTTTTTCTTCCACCAATGGAAGCAATGCACGCAGAGTCGACT
TGATATCGCCGACCAGTGCCTGGTAACACGACAACCGAAACGCCACGGTTAAGCACCGCTTTGCGCA
TGGCAAT

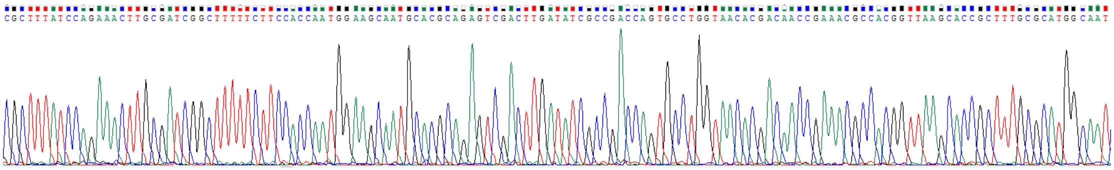

**_Poxb_ deletion using simple editing cassette construction strage:** 587bp was deleted from the *poxb* gene

GCTTTATCCAGAAACTTGCGATCGGCTTTTTCTTCCACCAATGGAAGCAATGCACGCAGAGTCGAAAT
AGCTCTTGTGGGTGGGTTTCCTGGAAATAGCCGCTGCCAATTTCGCTGGAGGGAATATGAGCGGCA
ATCG

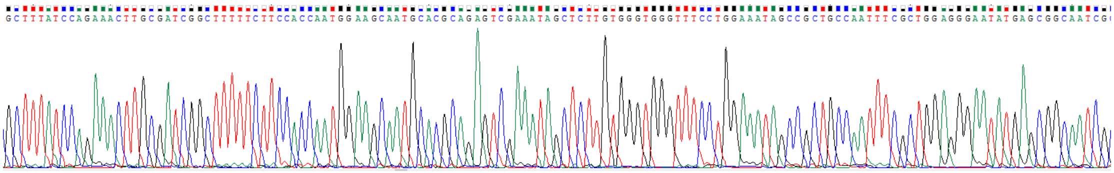

**_Lacz_ replaced by _kan_:** 1018bp of *lacz* was replaced by 915 bp fragment derived from the *kan* gene

Forward

AGGACAGTCGTTTGCCGTCTGAATTTGACCTGAGCGCATTTTTAGGCGCCGGAGAAAACCGCTCGTC
GGAATTGCCAGCTGGGGCGCCCTCTGGTAAGGTTGGGAAGCCCTGCAAAGTAAACTGGATGGCTTT
CTTGCCGCCAA

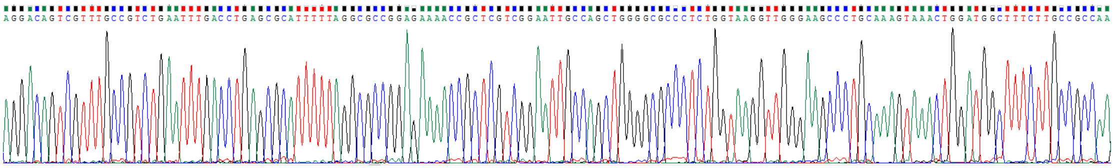

Reverse

AGACTGTTACCCATCGCGTGGGCGTATTCGCAAAGGATCAGCGGGCGCGTCTCTCCAGGTAGCGAA
AGCCAGTCTCAGAAGAACTCGTCAAGAAGGCGATAGAAGGCGATGCGCTGCGAATCGGGAGCGGC
GATACCGT

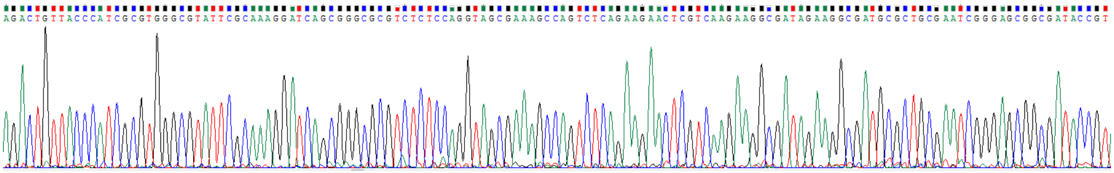

*Poxb* replaced by *kan*: 443 bp of *poxb* was replaced by 915 bp fragment derived from the *kan* gene

Forward

ATCCCACAAGTACTGGCGATTGCCATGCGCAAAGCGGTGCTTAACCGTGGCGTTTCGGTTGTCGTGT
TACCAGGCCGTCGGAATTGCCAGCTGGGGCGCCCTCTGGTAAGGTTGGGAAGCCCTGC

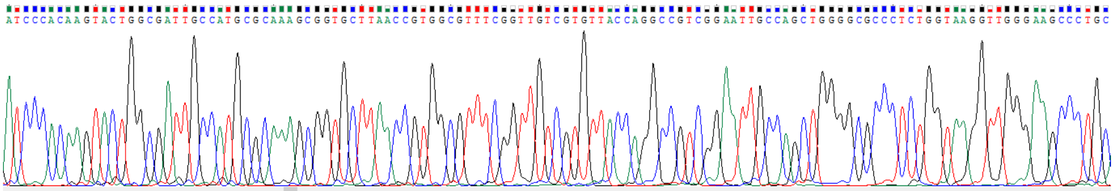

Reverse

CCAGCGCTTTATCCAGAAACTTGCGATCGGCTTTTTCTTCCACCAATGGAAGCAATGCACGCAGAGTC
GACTTGATATCGCCGACCAGTAGTCTCAGAAGAACTCGTCAAGAAGGCGATAGAAGGCGATGCGCT
GCG

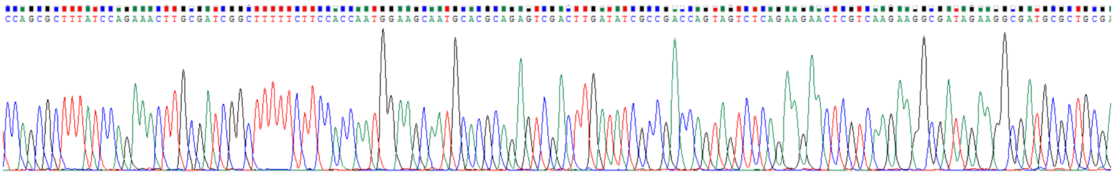

**pCAGO Sequence**

>

CACAACCGGCACGGAACTCGCTCGGGCTGGCCCCGGTGCATTTTTTAAATACCCG
CGAGAAATAGAGTTGATCGTCAAAACCAACATTGCGACCGACGGTGGCGATAGG
CATCCGGGTGGTGCTCAAAAGCAGCTTCGCCTGGCTGATACGTTGGTCCTCGCGC
CAGCTTAAGACGCTAATCCCTAACTGCTGGCGGAAAAGATGTGACAGACGCGAC
GGCGACAAGCAAACATGCTGTGCGACGCTGGCGATATCAAAATTGCTGTCTGCCA
GGTGATCGCTGATGTACTGACAAGCCTCGCGTACCCGATTATCCATCGGTGGATG
GAGCGACTCGTTAATCGCTTCCATGCGCCGCAGTAACAATTGCTCAAGCAGATTT
ATCGCCAGCAGCTCCGAATAGCGCCCTTCCCCTTGCCCGGCGTTAATGATTTGCC
CAAACAGGTCGCTGAAATGCGGCTGGTGCGCTTCATCCGGGCGAAAGAACCCCG
TATTGGCAAATATTGACGGCCAGTTAAGCCATTCATGCCAGTAGGCGCGCGGACG
AAAGTAAACCCACTGGTGATACCATTCGCGAGCCTCCGGATGACGACCGTAGTG
ATGAATCTCTCCTGGCGGGAACAGCAAAATATCACCCGGTCGGCAAACAAATTCT
CGTCCCTGATTTTTCACCACCCCCTGACCGCGAATGGTGAGATTGAGAATATAAC
CTTTCATTCCCAGCGGTCGGTCGATAAAAAAATCGAGATAACCGTTGGCCTCAAT
CGGCGTTAAACCCGCCACCAGATGGGCATTAAACGAGTATCCCGGCAGCAGGGG
ATCATTTTGCGCTTCAGCCATACTTTTCATACTCCCGCCATTCAGAGAAGAAACCA
ATTGTCCATATTGCATCAGACATTGCCGTCACTGCGTCTTTTACTGGCTCTTCTCG
CTAACCAAACCGGTAACCCCGCTTATTAAAAGCATTCTGTAACAAAGCGGGACCA
AAGCCATGACAAAAACGCGTAACAAAAGTGTCTATAATCACGGCAGAAAAGTCC
ACATTGATTATTTGCACGGCGTCACACTTTGCTATGCCATAGCATTTTTATCCATA
AGATTAGCGGATCCTACCTGACGCTTTTTATCGCAACTCTCTACTGTTTCTCCATA
CCCGTTTTTTTGGGAATTCGAGCTCTAAGGAGGTTATAAAAAATGGATAAGAAAT
ACTCAATAGGCTTAGATATCGGCACAAATAGCGTCGGATGGGCGGTGATCACTG
ATGAATATAAGGTTCCGTCTAAAAAGTTCAAGGTTCTGGGAAATACAGACCGCCA
CAGTATCAAAAAAAATCTTATAGGGGCTCTTTTATTTGACAGTGGAGAGACAGCG
GAAGCGACTCGTCTCAAACGGACAGCTCGTAGAAGGTATACACGTCGGAAGAAT
CGTATTTGTTATCTACAGGAGATTTTTTCAAATGAGATGGCGAAAGTAGATGATA
GTTTCTTTCATCGACTTGAAGAGTCTTTTTTGGTGGAAGAAGACAAGAAGCATGA
ACGTCATCCTATTTTTGGAAATATAGTAGATGAAGTTGCTTATCATGAGAAATAT
CCAACTATCTATCATCTGCGAAAAAAATTGGTAGATTCTACTGATAAAGCGGATT
TGCGCTTAATCTATTTGGCCTTAGCGCATATGATTAAGTTTCGTGGTCATTTTTTG
ATTGAGGGAGATTTAAATCCTGATAATAGTGATGTGGACAAACTATTTATCCAGT
TGGTACAAACCTACAATCAATTATTTGAAGAAAACCCTATTAACGCAAGTGGAGT
AGATGCTAAAGCGATTCTTTCTGCACGATTGAGTAAATCAAGACGATTAGAAAAT
CTCATTGCTCAGCTCCCCGGTGAGAAGAAAAATGGCTTATTTGGGAATCTCATTG
CTTTGTCATTGGGTTTGACCCCTAATTTTAAATCAAATTTTGATTTGGCAGAAGAT

GCTAAATTACAGCTTTCAAAAGATACTTACGATGATGATTTAGATAATTTATTGG
CGCAAATTGGAGATCAATATGCTGATTTGTTTTTGGCAGCTAAGAATTTATCAGA
TGCTATTTTACTTTCAGATATCCTAAGAGTAAATACTGAAATAACTAAGGCTCCC
CTATCAGCTTCAATGATTAAACGCTACGATGAACATCATCAAGACTTGACTCTTTT
AAAAGCTTTAGTTCGACAACAACTTCCAGAAAAGTATAAAGAAATCTTTTTTGAT
CAATCAAAAAACGGATATGCAGGTTATATTGATGGGGGAGCTAGCCAAGAAGAA
TTTTATAAATTTATCAAACCAATTTTAGAAAAAATGGATGGTACTGAGGAATTAT
TGGTGAAACTAAATCGTGAAGATTTGCTGCGCAAGCAACGGACCTTTGACAACG
GCTCTATTCCCCATCAAATTCACTTGGGTGAGCTGCATGCTATTTTGAGAAGACA
AGAAGACTTTTATCCATTTTTAAAAGACAATCGTGAGAAGATTGAAAAAATCTTG
ACTTTTCGAATTCCTTATTATGTTGGTCCATTGGCGCGTGGCAATAGTCGTTTTGC
ATGGATGACTCGGAAGTCTGAAGAAACAATTACCCCATGGAATTTTGAAGAAGTT
GTCGATAAAGGTGCTTCAGCTCAATCATTTATTGAACGCATGACAAACTTTGATA
AAAATCTTCCAAATGAAAAAGTACTACCAAAACATAGTTTGCTTTATGAGTATTT
TACGGTTTATAACGAATTGACAAAGGTCAAATATGTTACTGAAGGAATGCGAAA
ACCAGCATTTCTTTCAGGTGAACAGAAGAAAGCCATTGTTGATTTACTCTTCAAA
ACAAATCGAAAAGTAACCGTTAAGCAATTAAAAGAAGATTATTTCAAAAAAATA
GAATGTTTTGATAGTGTTGAAATTTCAGGAGTTGAAGATAGATTTAATGCTTCATT
AGGTACCTACCATGATTTGCTAAAAATTATTAAAGATAAAGATTTTTTGGATAAT
GAAGAAAATGAAGATATCTTAGAGGATATTGTTTTAACATTGACCTTATTTGAAG
ATAGGGAGATGATTGAGGAAAGACTTAAAACATATGCTCACCTCTTTGATGATAA
GGTGATGAAACAGCTTAAACGTCGCCGTTATACTGGTTGGGGACGTTTGTCTCGA
AAATTGATTAATGGTATTAGGGATAAGCAATCTGGCAAAACAATATTAGATTTTT
TGAAATCAGATGGTTTTGCCAATCGCAATTTTATGCAGCTGATCCATGATGATAG
TTTGACATTTAAAGAAGACATTCAAAAAGCACAAGTGTCTGGACAAGGCGATAG
TTTACATGAACATATTGCAAATTTAGCTGGTAGCCCTGCTATTAAAAAAGGTATT
TTACAGACTGTAAAAGTTGTTGATGAATTGGTCAAAGTAATGGGGCGGCATAAGC
CAGAAAATATCGTTATTGAAATGGCACGTGAAAATCAGACAACTCAAAAGGGCC
AGAAAAATTCGCGAGAGCGTATGAAACGAATCGAAGAAGGTATCAAAGAATTAG
GAAGTCAGATTCTTAAAGAGCATCCTGTTGAAAATACTCAATTGCAAAATGAAAA
GCTCTATCTCTATTATCTCCAAAATGGAAGAGACATGTATGTGGACCAAGAATTA
GATATTAATCGTTTAAGTGATTATGATGTCGATCACATTGTTCCACAAAGTTTCCT
TAAAGACGATTCAATAGACAATAAGGTCTTAACGCGTTCTGATAAAAATCGTGGT
AAATCGGATAACGTTCCAAGTGAAGAAGTAGTCAAAAAGATGAAAAACTATTGG
AGACAACTTCTAAACGCCAAGTTAATCACTCAACGTAAGTTTGATAATTTAACGA
AAGCTGAACGTGGAGGTTTGAGTGAACTTGATAAAGCTGGTTTTATCAAACGCCA
ATTGGTTGAAACTCGCCAAATCACTAAGCATGTGGCACAAATTTTGGATAGTCGC
ATGAATACTAAATACGATGAAAATGATAAACTTATTCGAGAGGTTAAAGTGATTA
CCTTAAAATCTAAATTAGTTTCTGACTTCCGAAAAGATTTCCAATTCTATAAAGTA

CGTGAGATTAACAATTACCATCATGCCCATGATGCGTATCTAAATGCCGTCGTTG
GAACTGCTTTGATTAAGAAATATCCAAAACTTGAATCGGAGTTTGTCTATGGTGA
TTATAAAGTTTATGATGTTCGTAAAATGATTGCTAAGTCTGAGCAAGAAATAGGC
AAAGCAACCGCAAAATATTTCTTTTACTCTAATATCATGAACTTCTTCAAAACAG
AAATTACACTTGCAAATGGAGAGATTCGCAAACGCCCTCTAATCGAAACTAATGG
GGAAACTGGAGAAATTGTCTGGGATAAAGGGCGAGATTTTGCCACAGTGCGCAA
AGTATTGTCCATGCCCCAAGTCAATATTGTCAAGAAAACAGAAGTACAGACAGG
CGGATTCTCCAAGGAGTCAATTTTACCAAAAAGAAATTCGGACAAGCTTATTGCT
CGTAAAAAAGACTGGGATCCAAAAAAATATGGTGGTTTTGATAGTCCAACGGTA
GCTTATTCAGTCCTAGTGGTTGCTAAGGTGGAAAAAGGGAAATCGAAGAAGTTA
AAATCCGTTAAAGAGTTACTAGGGATCACAATTATGGAAAGAAGTTCCTTTGAAA
AAAATCCGATTGACTTTTTAGAAGCTAAAGGATATAAGGAAGTTAAAAAAGACT
TAATCATTAAACTACCTAAATATAGTCTTTTTGAGTTAGAAAACGGTCGTAAACG
GATGCTGGCTAGTGCCGGAGAATTACAAAAAGGAAATGAGCTGGCTCTGCCAAG
CAAATATGTGAATTTTTTATATTTAGCTAGTCATTATGAAAAGTTGAAGGGTAGT
CCAGAAGATAACGAACAAAAACAATTGTTTGTGGAGCAGCATAAGCATTATTTA
GATGAGATTATTGAGCAAATCAGTGAATTTTCTAAGCGTGTTATTTTAGCAGATG
CCAATTTAGATAAAGTTCTTAGTGCATATAACAAACATAGAGACAAACCAATACG
TGAACAAGCAGAAAATATTATTCATTTATTTACGTTGACGAATCTTGGAGCTCCC
GCTGCTTTTAAATATTTTGATACAACAATTGATCGTAAACGATATACGTCTACAA
AAGAAGTTTTAGATGCCACTCTTATCCATCAATCCATCACTGGTCTTTATGAAACA
CGCATTGATTTGAGTCAGCTAGGAGGTGACTGAGCATCAAATAAAACGAAAGGC
TCAGTCGAAAGACTGGGCCTTTCGTTTTATCTGTCTAGGTTTATACATAGGCGAGT
ACTCTGTTATGGAGTCAGATCTTAGCTAGTCCATCGAACCGAAGTAGTTTTAGAG
CTAGAAATAGCAAGTTAAAATAAGGCTAGTCCGTTATCAACTTGAAAAAGTGGC
ACCGAGTCGGTGCTTAGCATCCAAACTCGAGTAAGGATCTCCAGGCATCAAATAA
AACGAAAGGCTCAGTCGAAAGACTGGGCCTTTCGTTTTATCTGTTGTTTGTCGGT
GAACGCTCTCTACTAGAGTCACACTGGCTCACCTTCGGGTGGGCCTTTCTGCGTTT
ATACCTAGGGCGTTCGGCTGCGGCTCTACTTTTGTTTGTTAGTCTTGATGCTTCAC
TGATAGATACAAGAGCCATAAGAACCTCAGATCCTTCCGTATTTAGCCAGTATGT
TCTCTCCATACGATATAAGTTGTAATTCTCATGTTTGACAGCTTATCATCGTTAAT
TAACTAGTCATATGGGCATGCATTTACGTTGACACCATCGAATGGTGCAAAACCT
TTCGCGGTATGGCATGATAGCGCCCGGAAGAGAGTCAATTCAGGGTGGTGAATG
TGAAACCAGTAACGTTATACGATGTCGCAGAGTATGCCGGTGTCTCTTATCAGAC
CGTTTCCCGCGTGGTGAACCAGGCCAGCCACGTTTCTGCGAAAACGCGGGAAAA
AGTGGAAGCGGCGATGGCGGAGCTGAATTACATTCCCAACCGCGTGGCACAACA
ACTGGCGGGCAAACAGTCGTTGCTGATTGGCGTTGCCACCTCCAGTCTGGCCCTG
CACGCGCCGTCGCAAATTGTCGCGGCGATTAAATCTCGCGCCGATCAACTGGGTG
CCAGCGTGGTGGTGTCGATGGTAGAACGAAGCGGCGTCGAAGCCTGTAAAGCGG

CGGTGCACAATCTTCTCGCGCAACGCGTCAGTGGGCTGATCATTAACTATCCGCT
GGATGACCAGGATGCCATTGCTGTGGAAGCTGCCTGCACTAATGTTCCGGCGTTA
TTTCTTGATGTCTCTGACCAGACACCCATCAACAGTATTATTTTCTCCCATGAAGA
CGGTACGCGACTGGGCGTGGAGCATCTGGTCGCATTGGGTCACCAGCAAATCGC
GCTGTTAGCGGGCCCATTAAGTTCTGTCTCGGCGCGTCTGCGTCTGGCTGGCTGG
CATAAATATCTCACTCGCAATCAAATTCAGCCGATAGCGGAACGGGAAGGCGAC
TGGAGTGCCATGTCCGGTTTTCAACAAACCATGCAAATGCTGAATGAGGGCATCG
TTCCCACTGCGATGCTGGTTGCCAACGATCAGATGGCGCTGGGCGCAATGCGCGC
CATTACCGAGTCCGGGCTGCGCGTTGGTGCGGATATCTCGGTAGTGGGATACGAC
GATACCGAAGACAGCTCATGTTATATCCCGCCGTTAACCACCATCAAACAGGATT
TTCGCCTGCTGGGGCAAACCAGCGTGGACCGCTTGCTGCAACTCTCTCAGGGCCA
GGCGGTGAAGGGCAATCAGCTGTTGCCCGTCTCACTGGTGAAAAGAAAAACCAC
CCTGGCGCCCAATACGCAAACCGCCTCTCCCCGCGCGTTGGCCGATTCATTAATG
CAGCTGGCACGACAGGTTTCCCGACTGGAAAGCGGGCAGTGAGCGCAACGCAAT
TAATGTGAGTTAGCGCGAATTGATCTGGTTTGACAGCTTATCATCGACTGCACGG
TGCACCAATGCTTCTGGCGTCAGGCAGCCATCGGAAGCTGTGGTATGGCTGTGCA
GGTCGTAAATCACTGCATAATTCGTGTCGCTCAAGGCGCACTCCCGTTCTGGATA
ATGTTTTTTGCGCCGACATCATAACGGTTCTGGCAAATATTCTGAAATGAGCTGTT
GACAATTAATCATCCGGCTCGTATAATGTGTGGAATTGTGAGCGGATAACAATTT
CACACAGGAAACAGACCATGGAATTCAAGGAGATATACCATGGATATTAATACT
GAAACTGAGATCAAGCAAAAGCATTCACTAACCCCCTTTCCTGTTTTCCTAATCA
GCCCGGCATTTCGCGGGCGATATTTTCACAGCTATTTCAGGAGTTCAGCCATGAA
CGCTTATTACATTCAGGATCGTCTTGAGGCTCAGAGCTGGGCGCGTCACTACCAG
CAGCTCGCCCGTGAAGAGAAAGAGGCAGAACTGGCAGACGACATGGAAAAAGG
CCTGCCCCAGCACCTGTTTGAATCGCTATGCATCGATCATTTGCAACGCCACGGG
GCCAGCAAAAAATCCATTACCCGTGCGTTTGATGACGATGTTGAGTTTCAGGAGC
GCATGGCAGAACACATCCGGTACATGGTTGAAACCATTGCTCACCACCAGGTTGA
TATTGATTCAGAGGTATAAAACGAATGAGTACTGCACTCGCAACGCTGGCTGGGA
AGCTGGCTGAACGTGTCGGCATGGATTCTGTCGACCCACAGGAACTGATCACCAC
TCTTCGCCAGACGGCATTTAAAGGTGATGCCAGCGATGCGCAGTTCATCGCATTA
CTGATCGTTGCCAACCAGTACGGCCTTAATCCGTGGACGAAAGAAATTTACGCCT
TTCCTGATAAGCAGAATGGCATCGTTCCGGTGGTGGGCGTTGATGGCTGGTCCCG
CATCATCAATGAAAACCAGCAGTTTGATGGCATGGACTTTGAGCAGGACAATGA
ATCCTGTACATGCCGGATTTACCGCAAGGACCGTAATCATCCGATCTGCGTTACC
GAATGGATGGATGAATGCCGCCGCGAACCATTCAAAACTCGCGAAGGCAGAGAA
ATCACGGGGCCGTGGCAGTCGCATCCCAAACGGATGTTACGTCATAAAGCCATG
ATTCAGTGTGCCCGTCTGGCCTTCGGATTTGCTGGTATCTATGACAAGGATGAAG
CCGAGCGCATTGTCGAAAATACTGCATACACTGCAGAACGTCAGCCGGAACGCG
ACATCACTCCGGTTAACGATGAAACCATGCAGGAGATTAACACTCTGCTGATCGC

CCTGGATAAAACATGGGATGACGACTTATTGCCGCTCTGTTCCCAGATATTTCGC
CGCGACATTCGTGCATCGTCAGAACTGACACAGGCCGAAGCAGTAAAAGCTCTT
GGATTCCTGAAACAGAAAGCCGCAGAGCAGAAGGTGGCAGCATGACACCGGACA
TTATCCTGCAGCGTACCGGGATCGATGTGAGAGCTGTCGAACAGGGGGATGATG
CGTGGCACAAATTACGGCTCGGCGTCATCACCGCTTCAGAAGTTCACAACGTGAT
AGCAAAACCCCGCTCCGGAAAGAAGTGGCCTGACATGAAAATGTCCTACTTCCA
CACCCTGCTTGCTGAGGTTTGCACCGGTGTGGCTCCGGAAGTTAACGCTAAAGCA
CTGGCCTGGGGAAAACAGTACGAGAACGACGCCAGAACCCTGTTTGAATTCACTT
CCGGCGTGAATGTTACTGAATCCCCGATCATCTATCGCGACGAAAGTATGCGTAC
CGCCTGCTCTCCCGATGGTTTATGCAGTGACGGCAACGGCCTTGAACTGAAATGC
CCGTTTACCTCCCGGGATTTCATGAAGTTCCGGCTCGGTGGTTTCGAGGCCATAA
AGTCAGCTTACATGGCCCAGGTGCAGTACAGCATGTGGGTGACGCGAAAAAATG
CCTGGTACTTTGCCAACTATGACCCGCGTATGAAGCGTGAAGGCCTGCATTATGT
CGTGATTGAGCGGGATGAAAAGTACATGGCGAGTTTTGACGAGATCGTGCCGGA
GTTCATCGAAAAAATGGACGAGGCACTGGCTGAAATTGGTTTTGTATTTGGGGAG
CAATGGCGATGACGCATCCTCACGATAATATCCGGGTAGGCGCAATCACTTTCGT
CTACTCCGTTACAAAGCGAGGCTGGGTATTTCCCGGCCTTTCTGTTATCCGAAATC
CACTGAAAGCACAGCGGCTGGCTGAGGAGATAAATAATAAACGAGGGGCTGTAT
GCACAAAGCATCTTCTGTTGAGTTAAGAACGAGTATCGAGATGGCACATAGCCTT
GCTCAAATTGGAATCAGGTTTGTGCCAATACCAGTAGAAACAGACGAAGAATCC
ATGGGTATGGACAGTTTTCCCTTTGATATGTAACGGTGAACAGTTGTTCTACTTTT
GTTTGTTAGTCTTGATGCTTCACTGATAGATACAAGAGCCATAAGAACCTCAGAT
CCTTCCGTATTTAGCCAGTATGTTCTCTAGTGTGGTTCGTTGTTTTTGCGTGAGCC
ATGAGAACGAACCATTGAGATCATACTTACTTTGCATGTCACTCAAAAATTTTGC
CTCAAAACTGGTGAGCTGAATTTTTGCAGTTAAAGCATCGTGTAGTGTTTTTCTTA
GTCCGTTACGTAGGTAGGAATCTGATGTAATGGTTGTTGGTATTTTGTCACCATTC
ATTTTTATCTGGTTGTTCTCAAGTTCGGTTACGAGATCCATTTGTCTATCTAGTTCA
ACTTGGAAAATCAACGTATCAGTCGGGCGGCCTCGCTTATCAACCACCAATTTCA
TATTGCTGTAAGTGTTTAAATCTTTACTTATTGGTTTCAAAACCCATTGGTTAAGC
CTTTTAAACTCATGGTAGTTATTTTCAAGCATTAACATGAACTTAAATTCATCAAG
GCTAATCTCTATATTTGCCTTGTGAGTTTTCTTTTGTGTTAGTTCTTTTAATAACCA
CTCATAAATCCTCATAGAGTATTTGTTTTCAAAAGACTTAACATGTTCCAGATTAT
ATTTTATGAATTTTTTTAACTGGAAAAGATAAGGCAATATCTCTTCACTAAAAACT
AATTCTAATTTTTCGCTTGAGAACTTGGCATAGTTTGTCCACTGGAAAATCTCAAA
GCCTTTAACCAAAGGATTCCTGATTTCCACAGTTCTCGTCATCAGCTCTCTGGTTG
CTTTAGCTAATACACCATAAGCATTTTCCCTACTGATGTTCATCATCTGAGCGTAT
TGGTTATAAGTGAACGATACCGTCCGTTCTTTCCTTGTAGGGTTTTCAATCGTGGG
GTTGAGTAGTGCCACACAGCATAAAATTAGCTTGGTTTCATGCTCCGTTAAGTCA
TAGCGACTAATCGCTAGTTCATTTGCTTTGAAAACAACTAATTCAGACATACATC

TCAATTGGTCTAGGTGATTTTAATCACTATACCAATTGAGATGGGCTAGTCAATG
ATAATTACTAGTCCTTTTCCTTTGAGTTGTGGGTATCTGTAAATTCTGCTAGACCT
TTGCTGGAAAACTTGTAAATTCTGCTAGACCCTCTGTAAATTCCGCTAGACCTTTG
TGTGTTTTTTTTGTTTATATTCAAGTGGTTATAATTTATAGAATAAAGAAAGAATA
AAAAAAGATAAAAAGAATAGATCCCAGCCCTGTGTATAACTCACTACTTTAGTCA
GTTCCGCAGTATTACAAAAGGATGTCGCAAACGCTGTTTGCTCCTCTACAAAACA
GACCTTAAAACCCTAAAGGCTTAAGTAGCACCCTCGCAAGCTCGGTTGCGGCCGC
AATCGGGCAAATCGCTGAATATTCCTTTTGTCTCCGACCATCAGGCACCTGAGTC
GCTGTCTTTTTCGTGACATTCAGTTCGCTGCGCTCACGGCTCTGGCAGTGAATGGG
GGTAAATGGCACTACAGGCGCCTTTTATGGATTCATGCAAGGAAACTACCCATAA
TACAAGAAAAGCCCGTCACGGGCTTCTCAGGGCGTTTTATGGCGGGTCTGCTATG
TGGTGCTATCTGACTTTTTGCTGTTCAGCAGTTCCTGCCCTCTGATTTTCCAGTCTG
ACCACTTCGGATTATCCCGTGACAGGTCATTCAGACTGGCTAATGCACCCAGTAA
GGCAGCGGTATCATCAACGGGGTCTGACGCTCAGTGGAACGAAAACTCACGTTA
AGGGATTTTGGTCATGAGATTATCAAAAAGGATCTTCACCTAGATCCTTTTAAAT
TAAAAATGAAGTTTTAAATCAATCTAAAGTATATATGAGTAAACTTGGTCTGACA
GTTACCAATGCTTAATCAGTGAGGCACCTATCTCAGCGATCTGTCTATTTCGTTCA
TCCATAGTTGCCTGACTCCCCGTCGTGTAGATAACTACGATACGGGAGGGCTTAC
CATCTGGCCCCAGTGCTGCAATGATACCGCGAGACCCACGCTCACCGGCTCCAGA
TTTATCAGCAATAAACCAGCCAGCCGGAAGGGCCGAGCGCAGAAGTGGTCCTGC
AACTTTATCCGCCTCCATCCAGTCTATTAATTGTTGCCGGGAAGCTAGAGTAAGT
AGTTCGCCAGTTAATAGTTTGCGCAACGTTGTTGCCATTGCTACAGGCATCGTGG
TGTCACGCTCGTCGTTTGGTATGGCTTCATTCAGCTCCGGTTCCCAACGATCAAGG
CGAGTTACATGATCCCCCATGTTGTGCAAAAAAGCGGTTAGCTCCTTCGGTCCTC
CGATCGTTGTCAGAAGTAAGTTGGCCGCAGTGTTATCACTCATGGTTATGGCAGC
ACTGCATAATTCTCTTACTGTCATGCCATCCGTAAGATGCTTTTCTGTGACTGGTG
AGTACTCAACCAAGTCATTCTGAGAATAGTGTATGCGGCGACCGAGTTGCTCTTG
CCCGGCGTCAATACGGGATAATACCGCGCCACATAGCAGAACTTTAAAAGTGCTC
ATCATTGGAAAACGTTCTTCGGGGCGAAAACTCTCAAGGATCTTACCGCTGTTGA
GATCCAGTTCGATGTAACCCACTCGTGCACCCAACTGATCTTCAGCATCTTTTACT
TTCACCAGCGTTTCTGGGTGAGCAAAAACAGGAAGGCAAAATGCCGCAAAAAAG
GGAATAAGGGCGACACGGAAATGTTGAATACTCATACTCTTCCTTTTTCAATATT
ATTGAAGCATTTATCAGGGTTATTGTCTCATGAGCGGATACATATTTGAATGTATT
TAGAAAAATAAACAAATAGGGGTTCCGCGCACATTTCCCCGAAAAGTGCCACCT
GCATCGATTTATTATGACAACTTGACGGCTACATCATTCACTTTTTCTT
